# VCC-BPS: Vertical Collaborative Clustering using Bit Plane Slicing

**DOI:** 10.1371/journal.pone.0244691

**Published:** 2021-01-11

**Authors:** WAQAR ISHAQ, ELIYA BUYUKKAYA, MUSHTAQ ALI, ZAKIR KHAN

**Affiliations:** 1 Department of Telecommunication, Hazara University, Mansehra, KP, Pakistan; 2 Department of Computer Engineering, Kadir Has University, Istanbul, Turkey; 3 Department of Information Technology, Hazara University, Mansehra, KP, Pakistan; University of Glasgow, UNITED KINGDOM

## Abstract

The vertical collaborative clustering aims to unravel the hidden structure of data (similarity) among different sites, which will help data owners to make a smart decision without sharing actual data. For example, various hospitals located in different regions want to investigate the structure of common disease among people of different populations to identify latent causes without sharing actual data with other hospitals. Similarly, a chain of regional educational institutions wants to evaluate their students’ performance belonging to different regions based on common latent constructs. The available methods used for finding hidden structures are complicated and biased to perform collaboration in measuring similarity among multiple sites. This study proposes vertical collaborative clustering using a bit plane slicing approach (VCC-BPS), which is simple and unique with improved accuracy, manages collaboration among various data sites. The VCC-BPS transforms data from input space to code space, capturing maximum similarity locally and collaboratively at a particular bit plane. The findings of this study highlight the significance of those particular bits which fit the model in correctly classifying class labels locally and collaboratively. Thenceforth, the data owner appraises local and collaborative results to reach a better decision. The VCC-BPS is validated by Geyser, Skin and Iris datasets and its results are compared with the composite dataset. It is found that the VCC-BPS outperforms existing solutions with improved accuracy in term of purity and Davies-Boulding index to manage collaboration among different data sites. It also performs data compression by representing a large number of observations with a small number of data symbols.

## 1 Introduction

Understanding the data is a great concern by data owners (e.g. business owners, government and private institutions, individuals, etc.). That concern can be defined as the way we discern the behavior of the data. Having a dataset *A*, with number of observations *n* = {*n*_1_, *n*_2_….*n*_*n*_} that are measured by number of features *X* = {*x*_1_, *x*_2_….*x*_*m*_}, provokes an intensive task of finding answers for questions that can contribute to data owner benefits. In other words, how can we learn from the behavior of this dataset? Ultimately, to learn something, first, you must have a goal. If the goal is to predict an output value *y* for given dataset *A*, that can be done via decision procedure known as *h*: *X* → *Y* where *Y* = {*y*_1_, *y*_2_….*y*_*n*_}. In this case, *A* turns into a training dataset that helps to train a mathematical algorithm model “*SA*” where its output is to predict a value *y*. Straightforward, substitute an observation *n*_*j*_ measured value vector *X* = {*x*_1_, *x*_2_….*x*_*m*_} to model “*SA*” the output is a prediction of *y*_*j*_ value. This goal is known as a supervised learning approach. This approach can be easily evaluated by using the evaluation set or cross-validation technique to predict an output value of the model [1]. On the contrary, if the goal is not to predict an output value of *y*, but to disclose possible hidden structure in dataset *A*, then such an approach would be known as unsupervised learning. The mathematical algorithm of this approach can find the type of underlying structure that the user has established either directly or indirectly in their approach. Sometimes, besides, the approach also provides some level of significance of the discovered structure. *Clustering* as a type of unsupervised learning approach, segregates observations into groups, called clusters, which may be mutually exclusive or overlap, relying on the technique used. The observations within a cluster are more similar to each other than the observations from another cluster. The similarity measure is of outstanding importance to define clusters that can be disclosed in the data. Different types of distances have been introduced in the literature with respect to the problem and context of the study [2].

**Fig 1:**
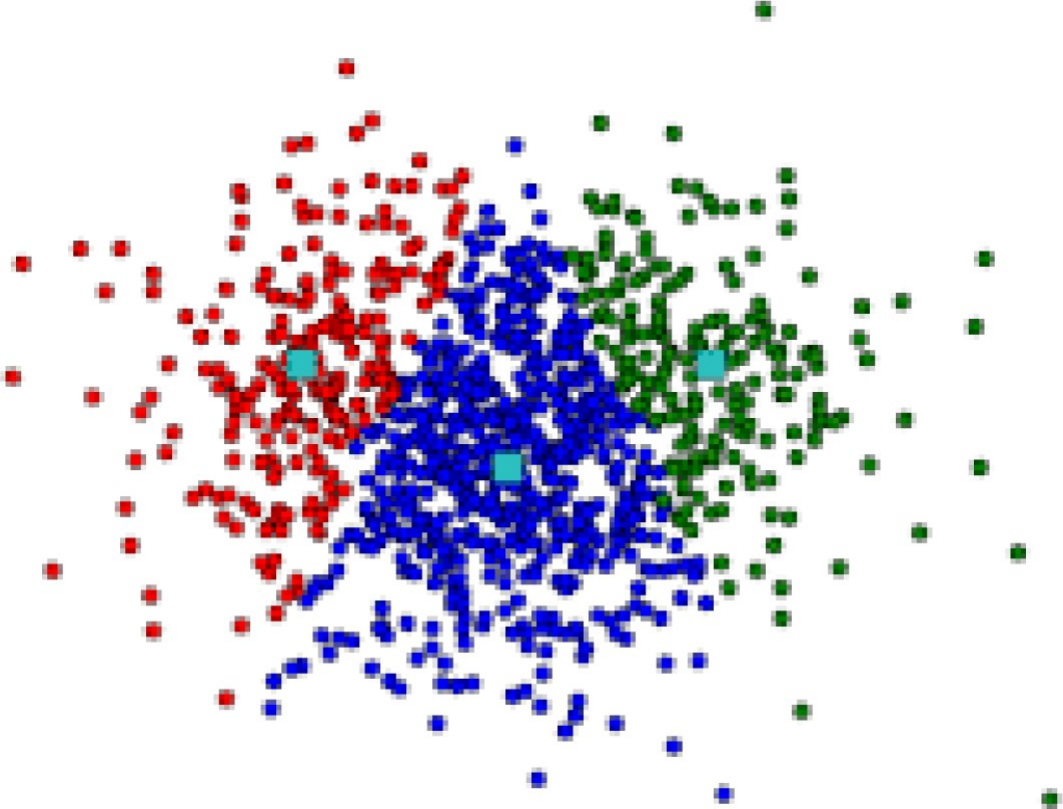
An example of clustering.

Supposing that the owner of the dataset *A* has the goal of using an unsupervised learning approach. For the sake of argument, let’s assume that the owner has essentially adhered to some criteria before adopting the approach, criteria such as defining the type of cluster will be looked for, organizing search space, validation methods (all of which will be introduced in the literature section). As a result of applying the approach, Fig 1 illustrates the clustering result of the dataset, represented in two dimensions. This result of clustering has been reached using {*x*_1_, *x*_2_, …, *x*_*m*_} of features on {*n*_1_, *n*_2_, *n*_3_, …, *n*_*n*_} of observations where m and n represent number of features and observations respectively for dataset *A*.

The question is with same number of features {*x*_1_, *x*_2_, …, *x*_*m*_}, however added a greater number of observations {*n*_1_, *n*_2_, *n*_3_, …, *n*_*n*+1_, *n*_*n*+2_, *n*_*n*+*n*_}, will the result of clustering remain the same? Will it be any better or worse? Another scenario, if the features have changed to a greater number, with the same observations, will the result again remain the same? Or even if both features and observations have changed, the same questions apply. Learning the behavior of 100 observations can help to discover a pattern. However, if the number grows to 1000 or more, positively the pattern would be more intelligent. To this end, a concept of “combining the clustering” is introduced. According to “combining the clustering” approach, the same clustering method can be applied over two or more datasets, then results are shared and merged to associate clusters of one site with other sites to identify similarity. This requires the selection of suitable clustering method(s), an adjustment in parameter values, number of features and observations, etc., to obtain unbiased outputs. This approach adds parallelization, scalability and robustness to the desired solution [3, 4]. One of combining the clustering inspiration ideas is known as collaborative clustering.

Let us assume that the owners of dataset A and B, having the same features, apply the same clustering algorithm and obtain local results *R*_*A*_ and *R*_*B*_ respectively. Now, is there any way that both owners can exchange information about the two results *R*_*A*_ and *R*_*B*_? If that is possible, then both owners will evaluate the final clustering result obtained from other sites in addition to their local data. The benefit here is augmenting the learning process of clustering local data through external clustering information of other sites. Technically speaking, different approaches are introduced to implement the idea, one of which is known as *horizontal* collaborative clustering (HCC) and the other is known as *vertical* collaborative clustering (VCC). In HCC approach, different datasets have same observations with different features, while in VCC approach, different datasets contain same features with different observations collaborate the clustering results [5, 6].

Different researchers worked on both of these approaches to explore hidden information and measure similarity among various independent datasets. This study focuses on vertical collaborative clustering by considering two independent organizations (*A* and *B*) having the same data features instead of one single tall dataset (combined dataset with a large number of observations) while maintaining data confidentiality. The vertical collaborative clustering using self-organizing mapping (SOM) [7, 8] and generative topographic mapping (GTM) [9–12] are existing approaches to apprehend data information among different data sites but have certain limitations which are mentioned as under:

1. SOM is sensitive to learning rate and neighborhood function in generating results which affects similarity measurement [13]. In SOM, all results rely on size of the map and a collaborative matrix which consists of collaborative coefficients, determines strength of each collaborative link, and degrade results if not set correctly [7, 14, 15]. Moreover, it lacks simplicity in calculating coefficients, which affects accuracy and performance. For further reading on SOM (see e.g. [16, 17])
2. GTM is non-linear approach of unsupervised learning and more precise than linear approaches but has higher run time complexity than linear approaches [10, 14]. GTM uses likelihood function for fast convergence and better tuning of topographic map parameters, which may not guarantee local convergence for all algorithms. Moreover, fast convergence does not ensure results of good quality [18, 19].
3. Accuracy of existing solutions is not verified by comparison of local and collaborative purity results with global purity for which datasets are pooled (all datasets are combined). Additionally, test data results are not mentioned to evaluate model generalization.

To overcome the above-mentioned limitations, this study proposes *the vertical collaborative clustering using bit plane slicing* (VCC-BPS) approach which is simple, accurate, and compresses data, managing collaboration among different data sites. The VCC-BPS consists of two phases i.e. local and collaborative phase to find a bit plane at which model fits the data to capture maximum similarity locally and collaboratively. The working principle of this approach is described as follows:

1. *Local Phase*: The object of the local phase is to look for that specific bit plane at which observations are grouped based on maximum similarity within the local dataset. It is an iterative process, searching for a bit plane at which code map fits the model in capturing maximum similarity locally.
2. *Collaborative Phase*: In a collaborative phase, the aim is to merge the local similarity of one dataset with that of others to produce collaborative results (collaborative similarity) similar to global similarity. This is achieved by merging the local result table of participating sites concerning common bit plane shared among them to identify maximum similarity. Finally, the data owner decides whether collaboration brings any new insight to uncover hidden information (similarity). The local and collaborative similarity is evaluated by purity and David-Bouldin index.

This study contributes a simple novel approach with improved accuracy in a match to existing approaches. The VCC-BPS can be used as a tool by different organizations and also performs compression to represent a large number of observations by small data codes to make smart decisions without compromising data confidentiality. Additionally, it produces collaborative results close to as if obtained from the pooled dataset (all datasets are combined).

The rest of the paper is organized as follows. In section 2, clustering, collaborative clustering with its requirements, types, and importance are explained. Section 3 elaborates the proposed methodology in detail. Section 4 mentions datasets used, evaluation metrics, and experimental results. Section 5 includes discussion and inferences. Section 6 mentions conclusion and future work.

## 2 Literature review

Since we propose Vertical Collaborative Clustering using the Bit Plane Slicing approach, this section briefly explains clustering, collaborative clustering with its requirements, types, importance, and bit plane slicing.

### 2.1 Clustering

The goal of clustering as a type of unsupervised learning, is to group clusters of observations that can be mutually exclusive or overlapped. The similarity is an important factor to decide the observations that are grouped together. Two approaches are mostly known for clustering [20]:

1. *Generative* approach is often based on statistical model, where the objective is to determine parameters that maximize how well the model fits the data.
2. *Discriminative* approach mostly depends on optimization criteria and similarity measurements to group the data.

Let us consider a buzzword known as ill-defined problem [21, 22]. This problem is considered from the idea that mathematically, the similarity is not a transitive relation while belonging to the same cluster. In other words, different methods may give inconsistent clustering outputs for the same data. Moreover, proper heuristics be employed to manage the computational cost. The following points need to be considered before adopting clustering approaches:

1. The first thing is to define the type of clusters being looked for, which relies on the context and our goal. The reason is that the same set of observations can be clustered in different ways, depending on type of distance used [23]. For example, measuring a distance between observations in the input space or between an observation and a cluster, may lead to a different clustering model [2].
2. The learning process of clustering is affected by the organization of the search space which is based on the number of variables, their degree of dependence, the type of normalization, etc. [5] discusses how the escalation of dimensionality increases the volume of the space exponentially.
3. The last matter is considering the validation step of the clustering model. Since there is no post validation method to compare true classes with the classes discovered by the algorithm for test data, clustering output evaluation is a delicate task. A few statistical procedures have been introduced to test the importance of the clustering result. They are based on measuring deviations of some statistical quantities but have certain limitations which create hindrances in getting the true findings [24].

### 2.2 Collaborative clustering and the vertical type

The clustering algorithms use two types of information during their computations:

1. Information about observation membership.
2. Information about internal parameters, such as the number of clusters anticipated, the coordinates of observations, and so on.

If we consider these two kinds of information developed from each local dataset, then the question is: Is it possible to exchange this information with another site that has a similarly structured dataset? The answer is “yes”. The concept, of doing so, is known as the collaborative clustering [25]. The goal is that the local clustering process can benefit from the work done by the other collaborator. In other words, collaborative clustering helps the local algorithm to escape from local minima (i.e. by only operating over a local dataset) by discovering better solutions (i.e. by exchanging information with another site that has a similar dataset). The validity is measured by the assumption that useful information be shared between the local sites. Important benefits of collaboration occur due to [5]:

1. Operating on local data in addition to information from other sites can help the algorithm to enhance the learning process.
2. The algorithm can escape local optima by using external information to get better solutions.
3. The local bias can be managed by using external information. However, this information can also be subject to other types of bias.

The core of this approach is accomplished by the exchange of information. Here information can be about the local data, or current hypothesized local clustering, or the value of one algorithm’s parameters. In other words, what can be shared between experts is information about data (e.g. features found useful, distances used, etc.) or information on the observation itself, such as the characteristic of an observation measured by a fixed feature vector. Important to mention is the performance measurement in clustering. It is hard to introduce an answer to such a question or in other words, no perfect answer can be reached. Therefore, there is no specific way of measuring the absolute quality of partitioning the data points. However, as mentioned above in the validity line that the assumption always lays on as the useful information is shared between the local sites. Nonetheless, some measurements are still a pioneer metric to measure the performance or the validity of the cluster. For example, [26] introduces a technique by defining the similarity between input clusters based on the graph structure. Notable, that the way the cluster is viewed can be a good matter of measuring the performance or the validity of the cluster. But still, as the goal is to look for a common structure among different datasets, it is no longer possible to make direct comparisons at the level of the observation since they are different. Only descriptions of the clusters found by the local algorithm can be exchanged, and a consensus measure must be defined at this level [27].

Following the above paragraph, it is important to discuss an important question that is how to control the collaboration phase? There are different approaches introduced in this domain, here are some of them [28]:

1. Synchronous or asynchronous operations: The former occurs when each local clustering process has its own goal and exchanges information only in the search of its local goal. The latter one is generally needed when the result depends on all local achievements.
2. Iterative or one-time process: The former occurs when the computations performed by each local algorithm can consider partial solutions shared by other algorithm sites and is therefore iterative. In a one-time process, all algorithms compute their local solution, after which a master algorithm combines them and outputs the final solution.
3. Local or global control: The former works with an asynchronous control strategy, while the latter is linked with the computation of a final combined overall solution [18].

However, regardless of the method of controlling, termination condition is of main concern in collaborative approaches. Where it is required that a clustering algorithm stops when a condition is met, even though the solution obtained might not be meeting the global optimization criterion [18].

To conclude this section, we will introduce the two most common types of implementing collaborative clustering. Noteworthy, other types are there, however, the focus of this paper is to discuss one type in particular. The two types known in collaborative clustering are [9]:

1. Horizontal collaborative clustering, the idea of it as the name may suggest, same observations, however, different features. In other words, let dataset *A* with set of observations {*n*_1_, *n*_2_….*n*_*n*_}, operates over the feature space {*x*_1_, *x*_2_….*x*_*m*_}. Another dataset with the same set of observations can be investigating again, however in different feature spaces such as {*z*_1_, *z*_2_….*z*_*m*_}. For detail on HCC, see e.g. [19, 29, 30].
2. Vertical collaborative clustering describes datasets in the same feature space but with different observations. In other words, let dataset A with set of observations {*n*_1_, *n*_2_….*n*_*n*_}, operates over the feature space {*x*_1_, *x*_2_….*x*_*m*_}. Another dataset B with different set of observations {*o*_1_, *o*_2_….*o*_*m*_}, however, operates in the same feature space {*x*_1_, *x*_2_….*x*_*m*_}.

The proposed work considers the last type of collaborative clustering which is the vertical collaborative clustering, has the following basic requirements [8]:

1. Type and number of features must be the same among data sites.
2. Share local findings with other sites, such that collaborative results obtained at each site are as if obtained from the pooled dataset (all datasets are combined).

Before closing this section, we may narrate some benefits of such an approach as following [8]:

1. Reduces time and space complexity.
2. Keeps data confidentiality.
3. Enhances scalability.

Recently [9, 12] proposed probabilistic approach of collaborative learning using generative topographic mapping (GTM) based on principles of vertical collaborative clustering to exchange the information for tunning the topographic maps parameters. [14] introduces nonlinear classification approach to interpolate missing data and performs nonlinear mapping between data and latent space using Generative Topographic Map (GTM). In [15], hybrid collaborative clustering approach which is a combination of vertical and horizontal collaborative clustering, use the Self Organizing Map (SOM) algorithm to find common structure by exchange of information. [31] explains collaborative classification among different information sources (data sites) with same features using SOM to reveal common structure of distributed data. Collaborative filtering makes use of available preference to predict unknown preferences based on clustering similarity measurement [32].

### 2.3 Bit Plane Slicing

**Fig 2:**
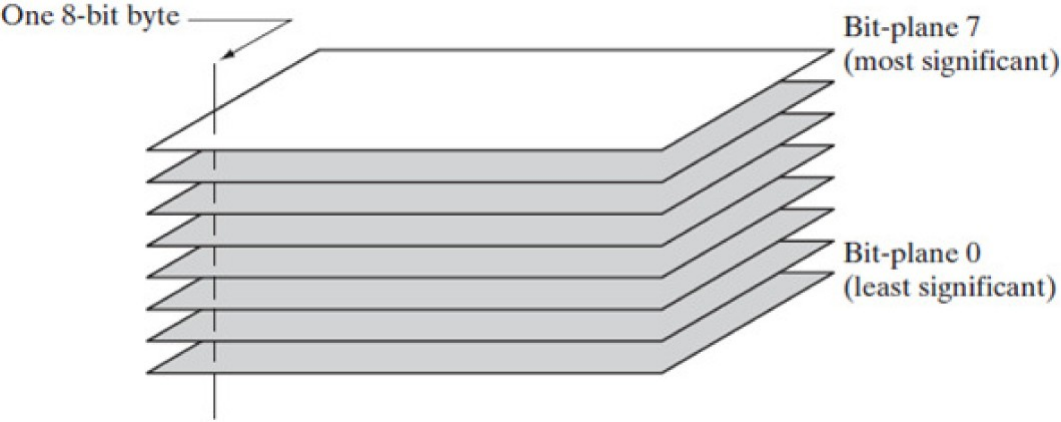
Bit Plane Slicing description [36].

The Bit Plane Slicing (BPS) is an image compression technique that divides a pixel of 8 bits image into 8-bit planes. Bit plane ranges from least significant bit (LSB) represented as bit-level 0 to most significant bit (MSB) marked as bit-level 7. The least and most significant bit plane contains all low and high order bits in the byte respectively. Change in low order bits of LSB does not change value much because they lack high contrast, while the change in high order bits of MSB signifies the change in data. Therefore, the most significant bit contains the majority of significant data and forms an image approximately similar to the original 8-bit image. This highlights the relative importance of specific bits in the image to reduce the image size. Based on such a strong characteristic of BPS, an 8-bit image containing a large amount of data is compressed into an image of small size with high similarity [33, 34]. The pictorial representation of bit plane slicing is shown in Fig 2 for an image composed of pixels, where each pixel occupies 8-bits memory and is represented by eight single-bit planes. The Eq (1) is used to form *k*^*th*^ bit plane with respect to *k*^*th*^ bit selected from all pixels. [35]:

$$\begin{array}{c} BitPlane_{k}=Reminder\left\{\frac{1}{2}floor\left[\frac{1}{2^{k}}Image\right]\right\} \end{array}$$ (1)

Where the value of k varies from 0 to 7. Suppose a gray scale image contains a pixel of intensity value 220. To find appropriate value for fourth bit plane, Eq (1) will return 1.

## 3 Proposed methodology

The prime reason for proposing vertical collaborative clustering using bit plane slicing, in addition to all benefits of using collaborative clustering, will enable a local data owner (e.g. business owners, government and private institutions, individuals, etc.) to find hidden structure in the process of implementing clustering techniques. This aim is logically explained since local data owner has the local capacity within the size of his/her data, however, enlarging the narratives that help to find hidden structure (in term of similarity among data sites without sharing data) by adding other information about clustering results from different sites, which happen to have same feature space with different observations, and can lead to better local clustering results by collaboration. For example, various hospitals located in different regions want to investigate the structure of common disease among people of different populations, identifying latent causes without sharing actual data with other hospitals. Similarly, a chain of regional educational institutes wants to evaluate their students’ performance belonging to different regions based on common latent constructs.

**Fig 3:**
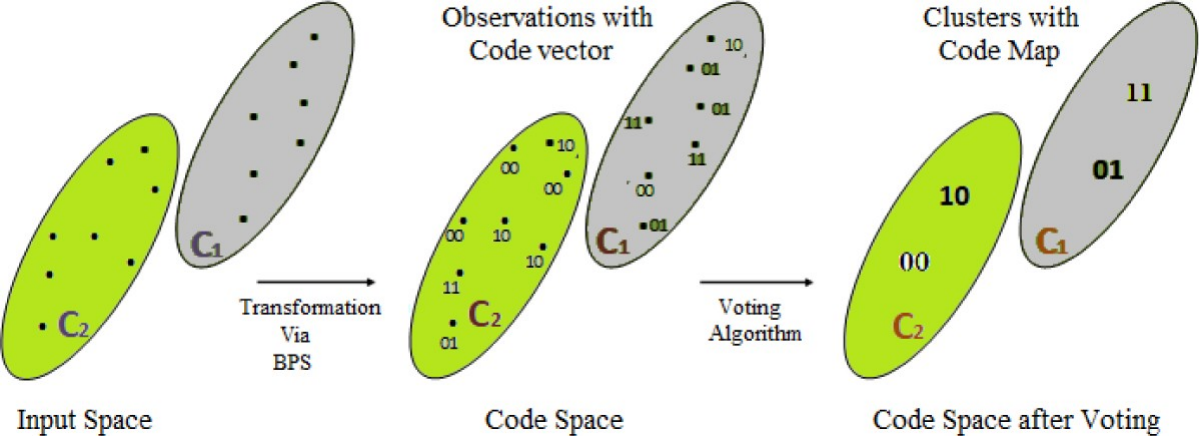
Transformation from input space to code space.

The proposed approach is termed as Vertical Collaborative Clustering using bit plane slicing (VCC-BPS), which performs collaboration among data sites where observations of similar code maps are associated with same class labels for common bit plane. In other words, mapping of all data inputs to a particular code map is done by searching for adequate common bit plane among sites where the model fits data with maximum similarity. Transformation from input space to code (latent) space is shown in Fig 3. The novelty of this approach is to capture not only similarity in local behavior but it also qualifies for collaboration to apprehend similarity among different datasets concerning common code space. This learning demands an unbiased environment where data of the same nature at different sites performs vertical collaborative clustering based on the following assumptions:

- Number of features and their type be the same (Requirement of VCC).
- Type of clustering method must be the same at all sites to avoid the influence of one clustering method over the other [4].
- Binary form after the decimal point is considered for computation.
- Bit plane consists of a single bit per feature to generate code.
- Common bit plane is selected for collaboration among data sites.
- Number of clusters be the same at all sites to deal with inconsistent output during collaboration [4].

**Fig 4:**
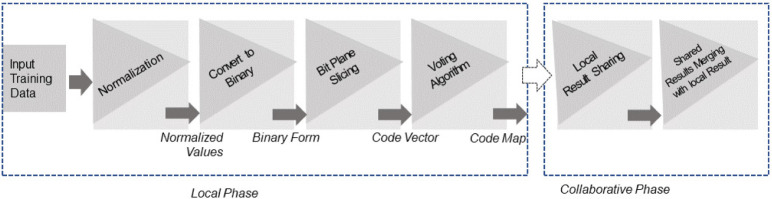
Proposed approach block diagram.

The vertical collaborative clustering using bit plane slicing consists of two phases i.e. local and collaborative phase to manage collaboration among data sites. The block diagram of the proposed approach is shown in Fig 4.

### 3.1 Local phase

According to the local phase, the dataset is first normalized to form common analytical plate form. The normalized value of each observation for given feature vector is converted into binary form. Then, BPS approach is used to compress feature vector into code vector and associated with corresponding class label for each observation. Finally, simple voting algorithm is applied over training dataset to predict class label for code vector called code map based on most frequent labels for selected bit plane. It captures data behavior where different observations for same class labels are encoded by same code map. In this phase, large volume of local data is compressed and represented as code map. Following are different steps involved in local phase:

1. Conversion to binary form and bit plane generation: In this step, dataset with features *X*_1_ and *X*_2_ is first normalized, having values between 0 and 1 and then converted to binary form as shown in column 4 (Binary form) of Table 1. Moreover, the observation value of each feature consists of 8 bits and represents 8 bit-plane. Bit plane is defined as a set of bits corresponding to the same bit position within observation in a data array (feature vector) shown in column 5 (Bit Plane 3 and 2) of Table 1.
2. Code vector generation: We are inspired by the Bit Plane Slicing approach [33, 34] which compresses image with high resemblance to the original one by considering the most significant bits. This study exploits such a strong characteristic of BPS when used with vertical collaborative clustering, highlights the relative importance of specific bits whether they are most or least significant bits or combination of both least and most significant bits in data, capturing maximum similarity. The purpose of using BPS is to transform binary input obtained from step 1 of the local phase into code space for a particular bit plane. According to BPS, a particular bit plane of one feature for each observation is concatenated to that of other features, forms code vector for given observation in the local dataset as shown in column 6 (Code Vector) of Table 1. The size of the code vector depends on the number of features. For example, number of features are two in a dataset, then number of bits per code(b) are two, assuming 1 bit per feature. Moreover, number of code vectors are *C*_*v*_ = 2^*b*^ = 2^2^ = 4 (00,01,10,11). Similarly, in case of four features, *C*_*v*_ = 2^4^ = 16. A code vector is a compressed form of actual data for a given observation at a particular bit plane.
3. Voting Algorithm: This step aims to correctly label the code vectors obtained from step 2 of the local phase. It is found in step 2 that certain observations have the same code vector mapping to different labels (clusters), thus forming the dual nature of the code vector. Notably, the same code vector must not belong to more than one class label or cluster. Such dual nature is shown in column 6 (Code Vector) versus column 7 (Class label) of Table 1. To solve such dual behavior, simple voting algorithm is used to find observations with a code vector of class label that repeats at least more than half of such observations, is considered in the majority. The voting algorithm helps to train the model to find code vector called code map for specific bit plane with the most frequent class labels (called predicted labels) as shown in column 8 (Code Map) and 9 (Predicted Label) of Table 1 respectively. Such mapping via simple voting algorithm correctly classifies class labels with least misclassification.
  The local phase of the proposed approach is an iterative approach to look for those bit planes in the local dataset at which observations are grouped based on maximum similarity in code space shown in Fig 3. Here, a search is made to determine a bit plane at which there is large contrast among observations to correctly group (cluster) them with least misclassification. For example, three observations with code vector 00 are labeled as cluster-2 (C-2) and one observation as cluster-1 (C-1) as shown in Fig 3, after transformation from input to code space using BPS. It is found in Table 1 that code vector 00 represents three observations {2, 6, 8} belonging to C-2 and one observation {15} belonging to C-1, thus reveals its dual behavior when bit plane is (3,2). Now in such scenario, using simple voting approach at particular bit plane (3,2) of feature *X*_1_ and *X*_2_ respectively, code vector 00 dominates C-2 in the match to C-1, therefore all observations in local dataset corresponding to code vector 00 are predicted or updated as class label C-2 (class in the majority). Moreover, observation {15} whose actual label is C-1 for code vector 00, is misclassified by the proposed approach. The same analogy is applied to other code vectors as shown in Table 1. It is important to mention that the same approach can be applied to the datasets with more than two clusters or class labels.
  In this phase, data is compressed to code map with most frequent class labels for selected bit planes. This forms the most important attribute of the proposed approach capturing not only similarity in local behavior but also qualifies for collaboration to apprehend similarity among different data sites for the same shared code space.

**Table 1:** Code map with predicted labels.

Obs.	Features Values		Normalized Values		Binary form		Bit Plan 3 and 2		Code Vector	Class Label	Code Map	Predicted Label
	*X*_1_	*X*_2_	*X*_1_	*X*_2_	*X*_1_	*X*_2_	*X*_1_	*X*_2_				
1	1.933	49	0.0951	0.1132	00001100	00001110	1	1	11	C-1	11	C-1
2	4.35	74	0.7857	0.5849	01100100	01001010	0	0	00	C-2	00	C-2
3	4.933	88	0.9523	0.8491	01111001	01101100	1	1	11	C-2	11	C-1
4	1.867	53	0.0763	0.1887	00001001	00011000	1	0	10	C-1	10	C-2
5	2.883	55	0.3666	0.2264	00101110	00011100	1	1	11	C-1	11	C-1
6	4.8	94	0.9143	0.9623	01110101	01111011	0	0	00	C-2	00	C-2
7	4.65	90	0.8714	0.8868	01101111	01110001	1	0	10	C-2	10	C-2
8	4	70	0.6857	0.5094	01010111	01000001	0	0	00	C-2	00	C-2
9	1.7	59	0.0286	0.3019	00000011	00100110	0	1	01	C-1	01	C-1
10	2.483	62	0.2523	0.3585	00100000	00101101	0	1	01	C-1	01	C-1
11	4.5	84	0.8286	0.7736	01101010	01100011	1	0	10	C-2	10	C-2
12	4.367	82	0.7906	0.7358	01100101	01011110	0	1	01	C-2	01	C-1
13	4.567	84	0.8477	0.7736	01101100	01100011	1	0	10	C-2	10	C-2
14	1.817	59	0.0620	0.3019	00000111	00100110	0	1	01	C-1	01	C-1
15	2.133	67	0.1523	0.4528	00010011	00111001	0	0	00	C-1	00	C-2

### 3.2 Collaborative phase

This phase aims to fulfill the basic requirement of vertical collaborative clustering, which is to share the local findings with other sites, such that collaborative results obtained at each site are as if obtained from pooled dataset [8]. This challenging task is addressed by the proposed approach, where similarities are identified among participating sites using following rules:

1. The same code map must represent the same class label among all participating sites at a particular bit plane. For example, code map 00 represents the class label C-1 at A with respect to particular bit plane, then the same code map must represent the same class label for the same bit plane at B.
2. There must be a common bit plane during the collaboration phase.
3. Only those local bit plane combinations are considered for collaboration that give local purity greater than 70% as threshold level.
4. More than one code map may represent the same class label locally. For example, at site A, code map 00 for an observation (*x*_1_), represents class label C-1. Similarly, code map 01 for other observations (*x*_2_), represents class label C-1 at A. It means both code maps fall in the same group labeled as C-1.

It is noticeable that those bit plane combinations where local results do not obey the above-mentioned rules, do not qualify for collaboration due to mismatch in behavior among participating sites. For example, observations with code map 00 represents the class label C-1 at A and the same code map represents observations with different class label (C-2) at B with respect to common bit plane e.g. (4,4). Then such bit plane combination in the light of the first rule do not qualify for collaboration due to mismatch in behavior among the participating sites. Likewise, if observations with code maps 00 and 01 represent the class label C-1 at site A and the code maps 00 and 10 represent observations with class label C-2 at site B with respect to common bit plane, then such bit plane is not considered for collaboration in light of the fourth rule.

In collaborative phase, the participating sites share their local results called the local result table. It consists of code vector with actual class label and code map with a predicted class label (code vector with the class label in the majority) for different bit plane combinations. When one data site local behavior matches with that of another data site in light of the above mentioned rules, then the collaborative purity is measured. The collaborative purity is computed as mean of local purities by merging shared results with local results at common bit plane combination such that local code map(s) matches with that of shared code maps. These rules ensure symmetry i.e. code map of one data site is exactly similar to another site with respect to common bit plane combinations. Such symmetry gives collaborative purity as the mean of all local purities with respect to common bit plane combinations among the participating sites where code map(s) at one site is similar to that at another site. Likewise, the collaborative DB index is measured by sharing local data cluster centroids and their variances among the participating sites under same rules.

## 4 Result evaluation

This section mentions datasets used, reason of their selection, evaluation metrics and experimental results.

### 4.1 Datasets

**Fig 5:**
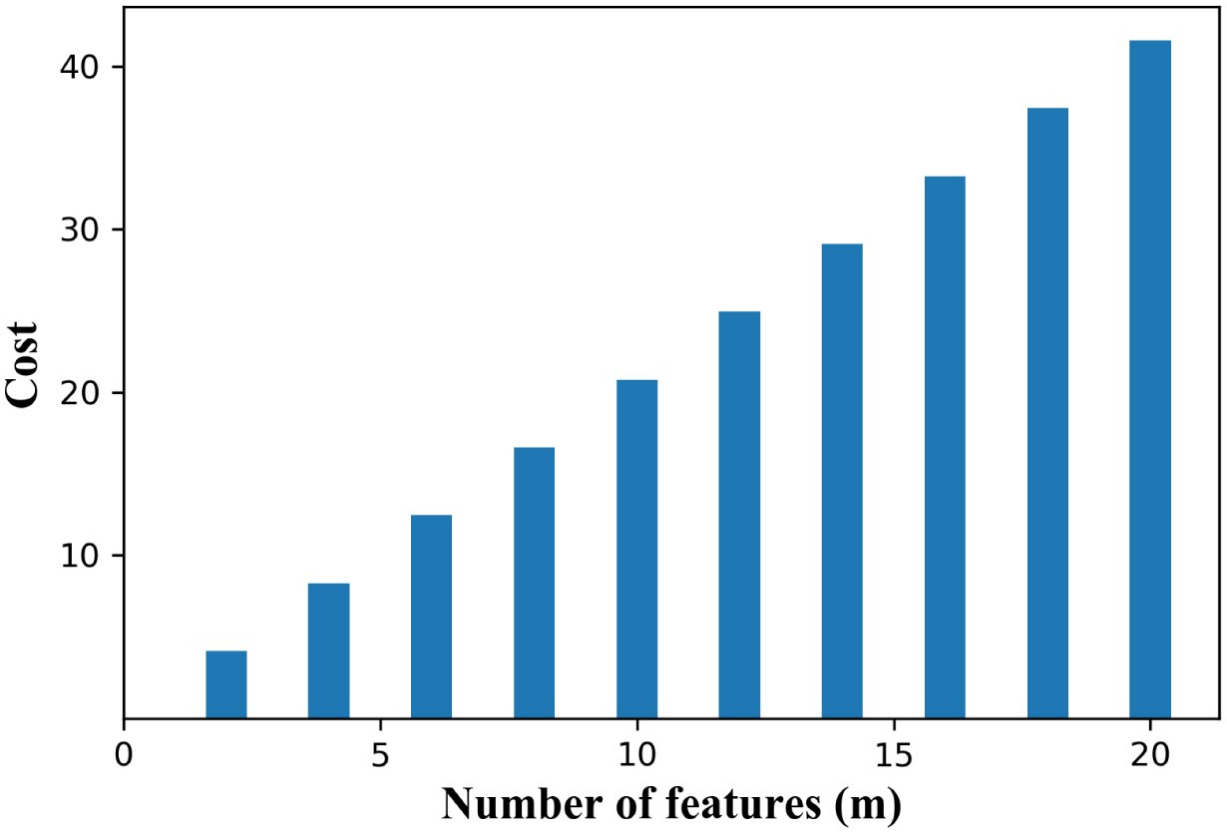
Computational cost of measuring purity.

To evaluate the proposed approach, three multivariate datasets i.e. Geyser [37], Skin segmentation (Skin) [38] and Iris [39] are used with features of real values. Skin dataset is tall dataset, consists of large number of observations with three features. To avoid computational complexity, these datasets of low dimensionality are chosen to explain and implement this novel approach simply and clearly. For example, a dataset with two features has 8-bit planes per feature and thus has (8^2^) 64-bit plane combinations for given feature space. Similarly, in the case of five features, search space for finding optimal solution consists of (8^5^) 32,768-bit plane combinations. This shows how much the search space explodes with the increase in number of features as shown in Fig 5. Therefore, datasets with small feature space are selected to reduce the search space to find a suitable bit plane.

To prepare the datasets for vertical collaborative clustering, having same features in a distributed environment (i.e. fulfilling the first requirement of VCC), the dataset is randomly divided into two data sites with same features, which are named as dataset *A* and *B* as shown in Table 2. Geyser dataset is subjected to the K-means algorithm for clustering/labeling the observations into two groups (i.e. C-1 and C-2) before BPS. The local and collaborative phases of the proposed approach are processed at each data site and then evaluated by purity and Davies-Bouldin index.

**Table 2:** Dataset description.

Dataset	# of Observations	# of Features	# of Class labels
Geyser [37]	136 × 2 sites	2	2 using K-mean
Skin [38]	122528 × 2 sites	3	2
Iris [39]	75 × 2 sites	4	3

### 4.2 Evaluation metrics

To evaluate the local results, the local purity is calculated based on their respective predicted and actual labels for given observations at a particular bit plane using Eq (2).

$$\begin{array}{c} P^{i}=\frac{1}{\left|n\right|}\sum_{k\in C}\underset{l\in L}{\text{max}}|c_{k}^{l}| \end{array}$$ (2)

Where *P*^*i*^ denotes local purity of the *i*^*th*^ data site, *n* and *C* refers to number of the observations and clusters respectively. *L* denotes the labels and $|c_{k}^{l}|$ describes the number of observations with label *l* in cluster *k*. The purity is the average proportion of the majority label in each cluster [6, 8]. The Eq (3) is used to compute the collaborative purity under certain rules (refer to section 3.2) to merge respective results.

$$\begin{array}{c} \bar{P}=\underset{C_{M}^{i}∼C_{M}^{j}}{Avg}{\left({\text{P}}^{i},{\text{P}}^{j}\right)}^{BP} \end{array}$$ (3)

**Fig 6:**
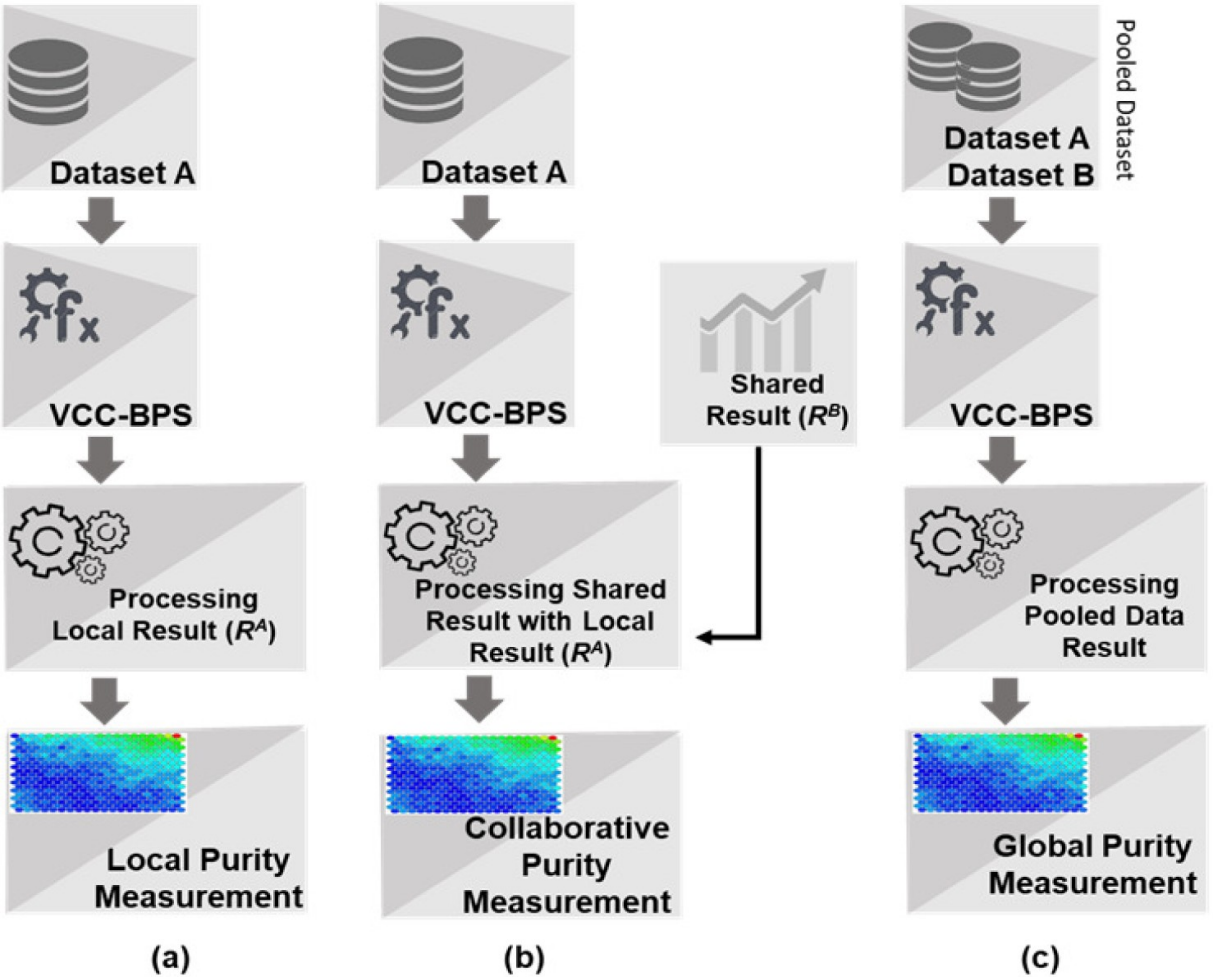
Process of local, collaborative and global purity measurements. (a) Local purity is computed at data site A without collaboration. (b) Collaborative purity is computed at A with respect to the result shared from site B, enhancing learning while data confidentiality is maintained. (c) Global purity is measured with respect pooled dataset where data confidentiality is compromised. This is done to check whether collaborative similarity is similar to global similarity.

Where $\bar{P}$ is collaborative purity, *P*^*i*^ and *P*^*j*^ denote local purities of the *i*^*th*^ and *j*^*th*^ data sites with respect to common bit plane combinations BP such that code map(s) at *i*^*th*^ data site ($C_{M}^{i}$) must be similar to that at *j*^*th*^ site. In addition to local and collaborative purity, the global purity is also used to evaluate the accuracy of collaborative outcome. For measuring the global purity, datasets are pooled and then purity is measured over the combined dataset clustering final map. The global purity with the local and collaborative purity is visually explained in Fig 6.

In addition to the purity as external index, Davies-Bouldin index is used as internal quality index to assess the compactness and separation of the resulting clusters [9] locally using Eq (4).

$$\begin{array}{c} DB=\frac{1}{K}\sum_{i=1}^{K}\underset{j\neq i}{\text{max}}\frac{S_{i}+S_{j}}{d(i,j)} \end{array}$$ (4)

Where *S*_*i*_ and *S*_*j*_ are local dispersions of *i*^*th*^ and *j*^*th*^ clusters, *d*(*i*, *j*) is centroid to centroid (inter cluster) distance for K number of given clusters using local dataset and *DB* refers to local Davies-Bouldin index. Local dispersion *S*_*i*_ and their corresponding inter-cluster distance, i.e. d(i,j) can be computed using Eqs (5) and (6).

$$\begin{array}{c} S_{i}=\frac{1}{T_{i}}\sum_{l=1}^{T_{i}}\|x_{l}-\mu_{i}{\|}^{2} \end{array}$$ (5)

$$\begin{array}{c} d\left(i,j\right)=\|\mu_{j}-\mu_{i}{\|}^{2} \end{array}$$ (6)

Where *x*_*l*_ is an observation in the dataset, associated with *i*^*th*^ cluster of size *T*_*i*_, having centroid *μ*_*i*_. Moreover, *μ*_*i*_ and *μ*_*j*_ refers to the centroid of the *i*^*th*^ and *j*^*th*^ cluster of same dataset (local dataset). The two clusters are considered similar, if they have large dispersion relative to their distance. Lower value of local DB indicates a cluster of better quality. Eq (6) is used to associate each cluster of dataset A with that of B to measure collaborative DB index:

$$\begin{array}{c} \bar{DB}=\frac{1}{K}\sum_{i=1}^{K}\underset{i,j\in K}{\text{max}}\frac{S_{i}^{A}+S_{j}^{B}}{D(i,j)}=\frac{1}{K}\sum_{i=1}^{K}\underset{i,j\in K}{\text{max}}\frac{S_{ij}^{AB}}{D(i,j)} \end{array}$$ (7)

Where $\bar{DB}$ is collaborative DB index, *D*(*i*, *j*) is the centroid to centroid distance between *i*^*th*^ and *j*^*th*^ cluster of dataset A and B respectively. Likewise, $S_{i}^{A}$ and $S_{j}^{B}$ are dispersions of *i*^*th*^ and *j*^*th*^ clusters of dataset A and B respectively. It is noticeable that low local DB value means observations within clusters are compact and clusters are well separated, whereas high collaborative DB value for dataset A and B means both have similarity in behavior and vice versa. In other words, Eq (4) reveals that local DB value is small when inter-cluster distance (*d*(*i*, *j*)) is large. Likewise, Eq (7) shows that collaborative DB value is large when inter cluster distance D(i,j) between clusters of A and B is small i.e. cluster i of A is similar to cluster j of B.

### 4.3 Experimental results

In this section, the local and collaborative results are evaluated by purity and DB index. It also presents the comparison of proposed approach (VCC-BPS) with existing approaches VCC-SOM [7] and VCC-GTM [9].

**Fig 7:**
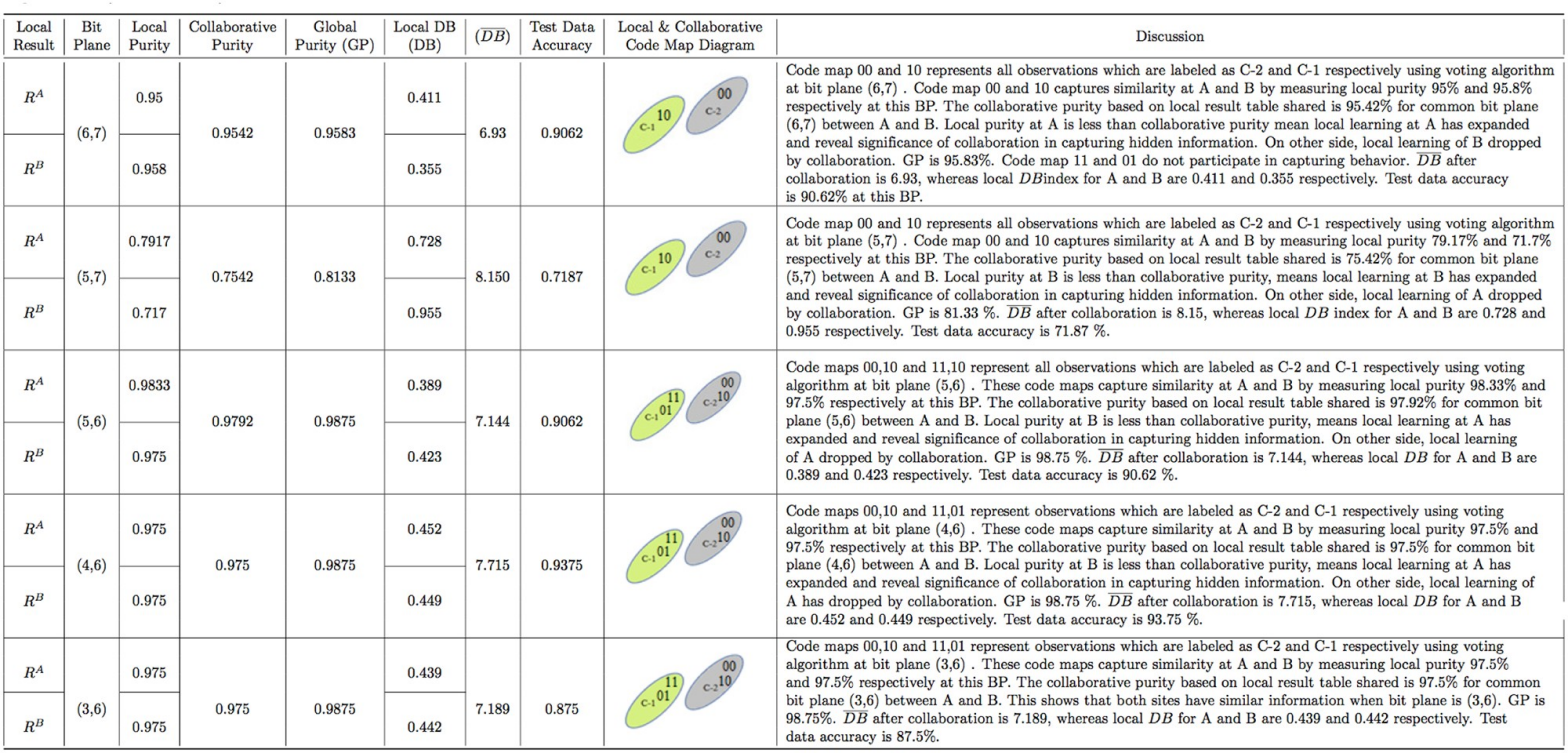
Geyser purity measurement and code map description.

In local phase of VCC-BPS, the normalized training datasets (*A* and *B*) of Geyser are converted into binary form and then subjected to BPS generating code vector, followed by simple voting algorithm to find code map with class labels in the majority at particular bit planes as shown in Table 3. The Table 3 consists of Geyser Data Local Result Table for site A and B, which explains that dataset A has 46 and 68 correctly classified observations belonging to class label C-2 and C-1, respectively, using simple voting algorithm with respect to bit plane (6,7). Similarly, the dataset B has 38 and 77 correctly classified observations belonging to class label C-2 and C-1, respectively at bit plane (6,7). The code maps 00 and 10 participate to associate observations with class C-2 and C-1 at A and B, respectively as shown in local and collaborative code map diagram column of Fig 7. Code maps 01 and 11 do not participate in capturing similarity locally at A and B when BP (6,7). The local purity is measured using Eq (2) for Geyser data at A and B, consisting of 120 training observations each as follows: Local purity at A = *P*^*A*^ = (46+68)/120 = 0.95 and *P*^*B*^ = (38+77)/120 = 0.958 such that code maps are 00 and 10 at both sites with BP (6,7). The detail about other bit plane combinations for Geyser data are shown in the discussion column of Table 3. The detail about Iris data having three classes, are mentioned in Table 4 with respect to only single bit plane combination (5,7,1,2) to avoid large computational local result table. The same analogy is applied to Skin datasets to generate local result table.

**Table 3:** Geyser dataset local result table for A and B (*R*^*A*^, *R*^*B*^) using purity index.

Bit Plane (BP)	Dataset (DS)	Cluster/Label	Code Vector				Code map				Discussion
			00	01	10	11	00	01	10	11	
(6,7)	DS-A	C-1	4	0	68	0	0	0	68	0	With BP (6,7) of features 1 and 2 respectively, the number of correctly classified observations at A and B belong to class label C-2 are 46 and 38, are represented by code map 00, whereas misclassified are 4 and 3 respectively. Likewise, 68 and 77 observations at A and B belong to label C-1, are represented by code map 10, whereas misclassified are 2 each respectively.
		C-2	46	0	2	0	46	0	0	0	
	DS-B	C-1	3	0	77	0	0	0	77	0	
		C-2	38	0	2	0	38	0	0	0	
(5,7)	DS-A	C-1	24	0	48	0	0	0	48	0	With BP (5,7), code map 00 receives 47 and 37 correctly classified votes to represent class label C-2 at A and B, whereas misclassified are 24 and 31 respectively. Similarly, code map 10 receives 48 and 49 votes to represent class label C-1 at A and B, whereas 1 and 3 are misclassified respectively.
		C-2	47	0	1	0	47	0	0	0	
	DS-B	C-1	31	0	49	0	0	0	49	0	
		C2	37	0	3	0	37	0	0	0	
(5,6)	DS-A	C-1	1	33	1	41	0	33	0	41	With BP (5,6), code map 00 receives 30 and 27 correctly classified votes to represent class label C-2 at A and B, whereas misclassified are 1 and 1 respectively. Likewise, code map 01 receives 33 and 35 correctly classified votes to represent class label C-1 at A and, B respectively. Similarly, code map 10 receives 14 and 13 votes to represent cluster C-2 at A and B respectively, whereas 1 and 2 are misclassified. Likewise, code map 11 has 41 and 42 correctly classified votes to represent C-1 at A and B respectively.
		C-2	30	0	14	0	30	0	14	0	
	DS-B	C-1	1	35	2	42	0	35	0	42	
		C-2	27	0	13	0	27	0	13	0	
(4,6)	DS-A	C-1	1	32	2	37	0	32	0	37	With BP (4,6), code map 00 receives 31 and 29 correctly classified votes to represent class label C-2 at A and B respectively, whereas 1 is misclassified at A. Likewise, code map 01 has 32 and 41 correctly classified votes representing C-1 at A and B respectively. Similarly, code map 10 receives 17 and 11 votes to represent C-2 at A and B, whereas 2 and 3 are misclassified respectively. Likewise, code map 11 has 37 and 36 correctly classified votes representing C-1 at A and B respectively.
		C-2	31	0	17	0	31	0	17	0	
	DS-B	C-1	0	41	3	36	0	41	0	36	
		C-2	29	0	11	0	29	0	11	0	
(3,6)	DS-A	C-1	3	34	0	35	0	34	0	35	With BP (3,6), code map 00 receives 23 and 22 correctly classified votes to represent cluster C-2 at A and B respectively, whereas 3 and 1 are misclassified. Likewise, code map 01 has 34 and 35 correctly classified votes representing C-1 at A and B respectively. Similarly, code map 10 receives 25 and 18 votes to represent cluster C-2 at A and B respectively, whereas 2 are misclassified at B. Likewise, code map 11 has 35 and 42 correctly classified votes representing C-1 at A and B respectively.
		C-2	23	0	25	0	23	0	25	0	
	DS-B	C-1	1	35	2	42	0	35	0	42	
		C-2	22	0	18	0	22	0	18	0	

**Table 4:** Iris dataset local result Table for A and B (*R*^*A*^, *R*^*B*^) using purity index.

Bit Plan	Data Set	Class Label	Code Vector								Code Map								Discussion
			0000	0001	0010	0011	1000	1001	1010	1011	0000	0001	0010	0011	1000	1001	1010	1011	
(5,7,1,2)	A	C-1	13	0	0	0	7	0	0	0	13	0	0	0	7	0	0	0	At BP (5,7,1,2), code map 0000 receives 13 and 9 correctly classified votes to represent cluster C-1 at A and B respectively, whereas 1 observation is misclassified at A. Likewise, code map 0001 has 2 and 4 correctly classified observations representing C-3, whereas misclassified are 1 each at A and B respectively. Similarly, code map 0010 has 8 and 5 correctly classified observations to represent class C-2, whereas misclassified observations are 4 and 2 at A and B respectively. Code map 0011 has 8 correctly classified observations each to represent class C-3, whereas misclassified observations are 3 and 2 at A and B respectively. Code map 1000 has 7 and 12 correctly classified observations to represent class C-1 at A and B respectively. Code map 1001 has 3 and 7 correctly classified observations to represent class C-2 at A and B respectively, whereas misclassified observations are 4 at B. Code Map 1010 has 7 and 4 correctly classified observations to represent class C-2, whereas 3 and 2 are misclassified observations at A and B respectively. Code map 1011 has 6 and 4 correctly classified observations to represent class C-3 at A and B respectively, whereas 2 are misclassified observations at B. The code maps not mentioned, are not involved in measuring similarity locally and collaboratively.
		C-2	1	1	8	3	0	3	7	0	0	0	8	0	0	3	7	0	
		C-3	0	2	4	8	0	0	3	6	0	2	0	8	0	0	0	6	
	B	C-1	9	0	0	0	12	0	0	0	9	0	0	0	12	0	0	0	
		C-2	0	1	5	2	0	7	4	2	0	0	5	0	0	7	4	0	
		C-3	0	4	2	8	0	4	2	4	0	4	0	8	0	0	0	4	

**Fig 8:**
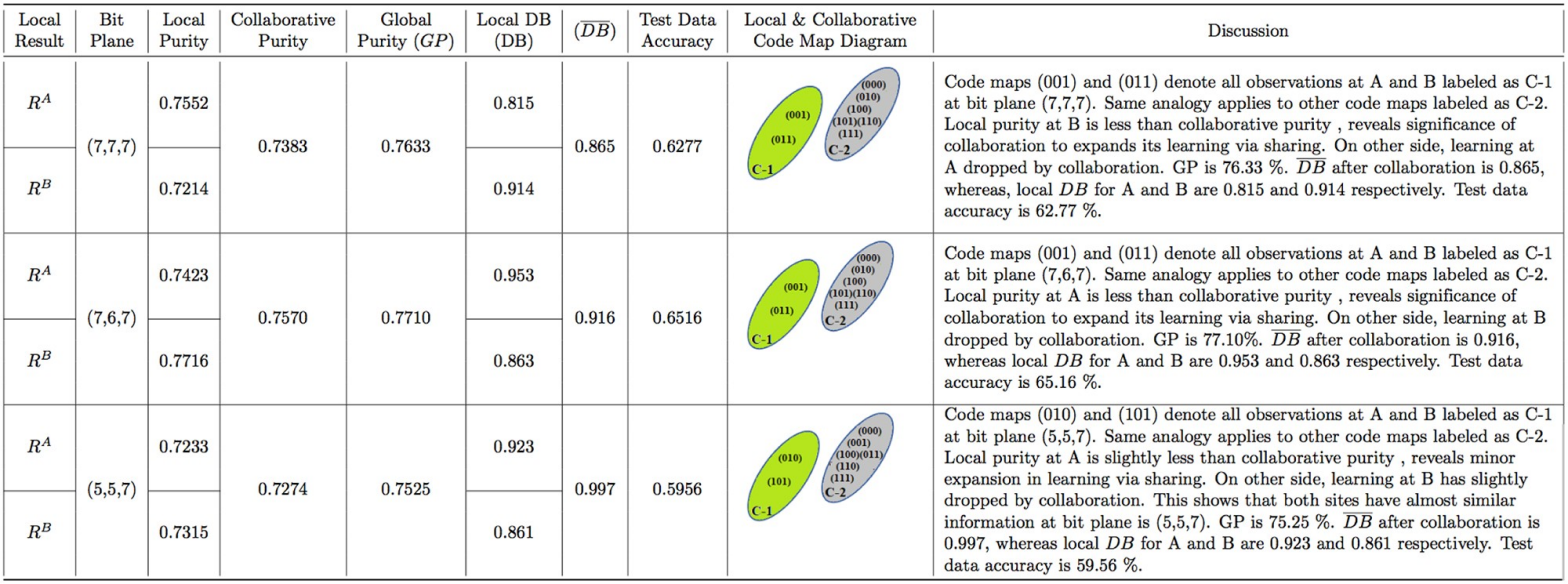
Skin purity measurement and code map description.

**Fig 9:**
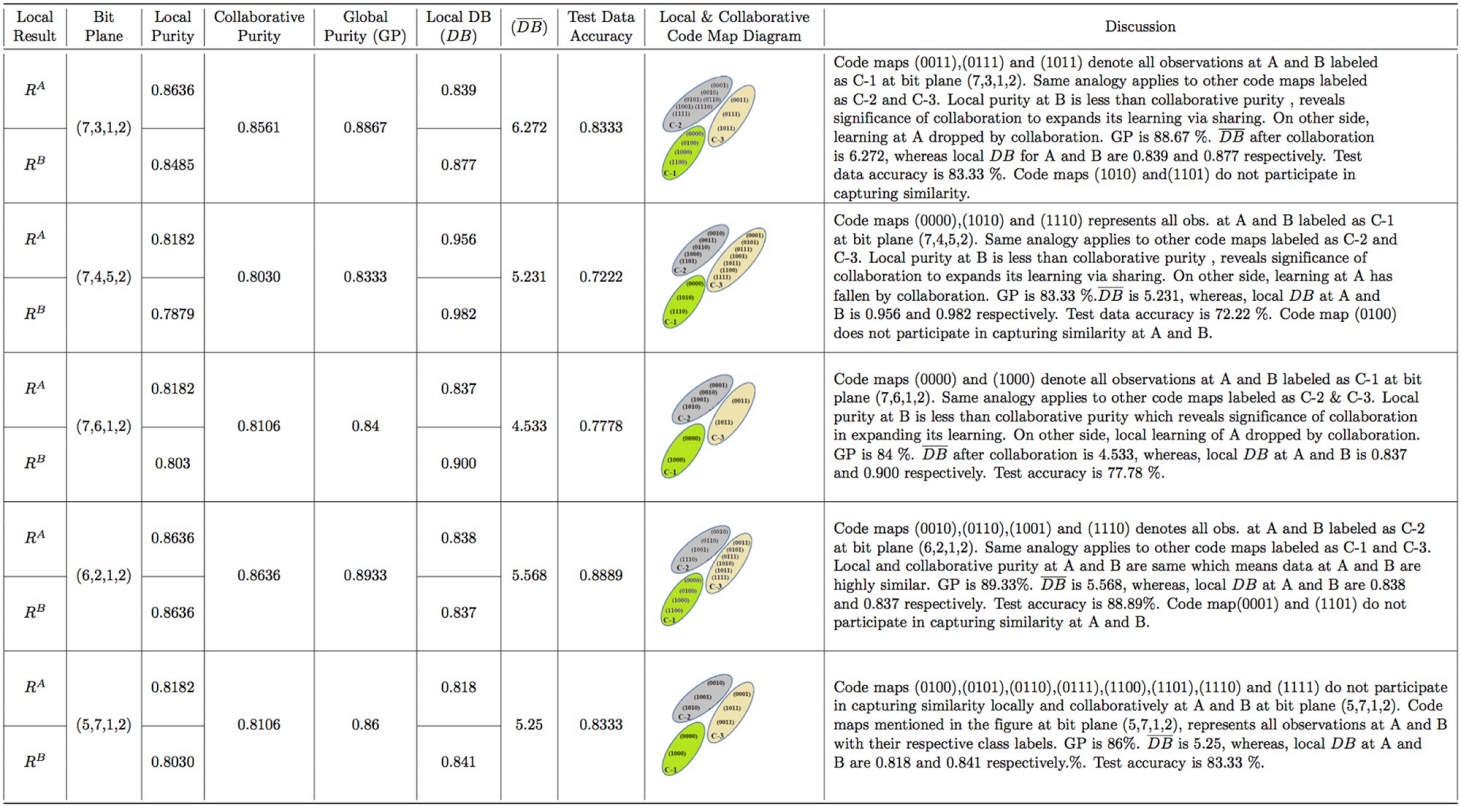
Iris purity measurement and code map description.

In collaborative phase of VCC-BPS, data site A and B of Geyser share their local result table (*R*^*A*^ and *R*^*B*^) and then collaborative purity is measured with respect to common bit plane as shown in Fig 7. The collaborative purity for Geyser data at site A and B is measured using Eq (3) as follows: $\bar{P}=\underset{C_{M}^{A}∼C_{M}^{B}}{Avg}{\left(P^{A},P^{B}\right)}^{BP}=\underset{00,10}{Avg}{\left(\left(46+68\right)/120,\left(38+77\right)/120\right)}^{\left(6,7\right)}=0.954$. The Fig 7 consists of the local, collaborative, global purity and DB indexes at site A and B with respect to particular bit plane for Geyser datasets. The same analogy is applied to Skin and Iris data consisting of 2 and 3 clusters with detail mentioned in discussion column of Figs 8 and 9.

The Davies-Bouldin index is used to evaluate the results of our proposed approach for Geyser, Skin and Iris data locally and collaborative at A and B. The local and collaborative DB index values are computed using Eqs (4) and (7), respectively. The details about computing local and collaborative DB for Iris data are mentioned in Tables 5 and 6. The same analogy is applied to Geyser and Skin data to measure their respective local and collaborative DB values as mentioned in Figs 7 and 8. To check the generalization of the proposed approach, test data is passed through the model and accuracy is determined for different bit planes as shown in Figs 7–9.

**Table 5:** Iris dataset local result table for A and B (*R*^*A*^, *R*^*B*^) using Davies Bouldin index.

Data site	Bit Plane	Cluster	Cluster Centroid				Local Dispersion (*S*_*i*_)	Local cluster to cluster distance d(i,j)			$\frac{S_{ij}}{d(i,j)}$			Local DB
								Cluster						
			X1	X2	X3	X4		1	2	3	1	2	3	
A	(7,3,1,2)	1	0.276	0.61	0.142	0.123	0.275	0.000	0.716	1.087	0	0.690	0.446	0.839
		2	0.46	0.304	0.599	0.543	0.219	0.716	0.000	0.470	0.690	0	0.913	
		3	0.696	0.460	0.820	0.846	0.210	1.087	0.470	0.000	0.446	0.913	0	
B		1	0.198	0.567	0.110	0.096	0.242	0	0.757	1.151	0	0.637	0.410	0.877
		2	0.419	0.284	0.584	0.564	0.240	0.757	0	0.471	0.637	0	0.997	
		3	0.733	0.385	0.815	0.809	0.230	1.151	0.471	0	0.410	0.997	0	
A	(7,4,5,2)	1	0.18	0.5960	0.075	0.051	0.136	0	0.674	1.147	0	0.673	0.324	0.956
		2	0.4370	0.3750	0.496	0.454	0.318	0.674	0	0.505	0.673	0	1.097	
		3	0.667	0.438	0.788	0.79	0.236	1.147	0.505	0	0.324	1.097	0	
B		1	0.144	0.56	0.073	0.062	0.176	0	0.681	1.140	0	0.678	0.410	0.982
		2	0.364	0.322	0.5	0.482	0.286	0.681	0	0.509	0.678	0	1.133	
		3	0.697	0.378	0.768	0.753	0.291	1.140	0.509	0	0.410	1.133	0	
A	(7,6,1,2)	1	0.2220	0.6330	0.072	0.059	0.163	0	0.788	1.140	0	0.464	0.350	0.837
		2	0.4730	0.3080	0.576	0.505	0.203	0.788	0	0.429	0.464	0	1.023	
		3	0.645	0.432	0.791	0.81	0.236	1.140	0.429	0	0.350	1.023	0	
B		1	0.155	0.563	0.074	0.063	0.173	0	0.770	1.161	0	0.565	0.351	0.900
		2	0.398	0.296	0.556	0.543	0.262	0.770	0	0.466	0.565	0	1.068	
		3	0.723	0.395	0.785	0.764	0.235	1.161	0.466	0	0.351	1.068	0	
A	(6,2,1,2)	1	0.2220	0.6330	0.072	0.059	0.163	0	0.813	1.134	0	0.466	0.345	0.838
		2	0.4360	0.2920	0.585	0.545	0.216	0.813	0	0.434	0.466	0	1.024	
		3	0.69	0.454	0.79	0.78	0.228	1.134	0.434	0	0.345	1.024	0	
B		1	0.161	0.579	0.074	0.063	0.117	0	0.784	1.122	0	0.418	0.306	0.837
		2	0.393	0.275	0.565	0.54	0.211	0.784	0	0.417	0.418	0	1.047	
		3	0.679	0.37	0.761	0.752	0.226	1.122	0.417	0	0.306	1.047	0	
A	(5,7,1,2)	1	0.2130	0.6230	0.074	0.06	0.156	0	0.775	1.114	0	0.465	0.350	0.818
		2	0.4200	0.3040	0.561	0.527	0.204	0.775	0	0.441	0.465	0	0.994	
		3	0.687	0.449	0.777	0.762	0.234	1.114	0.441	0	0.350	0.994	0	
B		1	0.161	0.579	0.074	0.063	0.184	0	0.764	1.105	0	0.509	0.399	0.841
		2	0.352	0.245	0.543	0.528	0.205	0.764	0	0.459	0.509	0	1.006	
		3	0.675	0.38	0.754	0.737	0.257	1.105	0.459	0	0.399	1.006	0	

**Table 6:** Iris Davies Bouldin measurement.

Bit Plane	Cluster		$S_{i}^{A}$	$S_{j}^{B}$	D(i,j)			$\frac{S_{ij}^{AB}}{D(i,j)}$			$\bar{DB}$
	A	B			Cluster						
	i	j			1	2	3	1	2	3	
(7,3,1,2)	1	1	0.275	0.242	0.098	0.719	1.088	5.253	0.717	0.464	6.272
	2	2	0.219	0.240	0.759	0.052	0.446	0.607	8.758	1.008	
	3	3	0.210	0.230	1.152	0.493	0.092	0.393	0.913	4.804	
(7,4,5,2)	1	1	0.136	0.176	0.052	0.689	1.135	5.986	0.612	0.376	5.231
	2	2	0.318	0.286	0.673	0.198	0.481	0.734	3.051	1.267	
	3	3	0.236	0.291	1.153	0.532	0.079	0.357	0.981	6.656	
(7,6,1,2)	1	1	0.163	0.173	0.097	0.783	1.146	3.464	0.543	0.347	4.533
	2	2	0.203	0.262	0.783	0.087	0.425	0.480	5.329	1.030	
	3	3	0.236	0.235	1.153	0.454	0.098	0.355	1.097	4.806	
(6,2,1,2)	1	1	0.163	0.117	0.082	0.795	0.795	3.432	0.471	0.489	5.568
	2	2	0.216	0.211	0.807	0.051	0.373	0.413	8.434	1.186	
	3	3	0.228	0.226	1.150	0.478	0.094	0.300	0.918	4.839	
(5,7,1,2)	1	1	0.156	0.184	0.068	0.775	1.092	4.987	0.466	0.378	5.250
	2	2	0.204	0.205	0.771	0.092	0.390	0.503	4.455	1.182	
	3	3	0.234	0.257	1.130	0.513	0.078	0.370	0.855	6.308	

The existing approaches which are VCC using SOM [7] and GTM [9] are implemented and tested over Geyser, Skin and Iris datasets for comparison with the proposed approach as shown in Table 7. The results of VCC-SOM and VCC-GTM approaches are topographic maps, representing compressed form of original dataset for given number of class labels mentioned in Table 2. Since SOM and GTM do not perform direct clustering, but are coupled with K-means approach and EM algorithm respectively over final map to extract clusters. Then purity and Davies-Bouldin index are measured over final map using Eqs (2) and (4) [9, 11, 12]. The size of the map is 5 × 5 for existing approaches to capture similar behavior among participating data sites. The Table 7 mentions local and collaborative results (i.e. collaboration of A with B and vice versa) for existing approaches using purity and DB index.

**Table 7:** Comparison of existing and proposed work.

Methods		Data site	Purity		Davies Bouldin Index	
			Local	Collaborative	DB	$\bar{DB}$
Existing	VCC-SOM [7]	*A*_*Geyser*_	93.38	94.85	0.546	0.531
		*B*_*Geyser*_	96.32	95.48	0.533	0.554
		*A*_*Skin*_	73.16	71.89	0.865	0.901
		*B*_*Skin*_	70.62	72.13	0.881	0.876
		*A*_*Iris*_	80	80	0.702	0.702
		*B*_*Iris*_	80	82.45	0.702	0.678
	VCC-GTM [9]	*A*_*Geyser*_	93.4	94.64	0.547	0.536
		*B*_*Geyser*_	95.88	94.23	0.541	0.567
		*A*_*Skin*_	74.64	72.77	0.872	0.875
		*B*_*Skin*_	70.91	73.12	0.88	0.866
		*A*_*Iris*_	84.3	85.17	0.712	0.701
		*B*_*Iris*_	86.04	84.29	0.668	0.691
Proposed	VCC-BPS at BP (5,6)	*A*_*Geyser*_	98.33	97.92	0.389	7.144
		*B*_*Geyser*_	97.5		0.423	
	VCC-BPS at BP (7,6,7)	*A*_*Skin*_	74.23	75.70	0.953	0.916
		*B*_*Skin*_	77.16		0.863	
	VCC-BPS at BP (6,2,1,2)	*A*_*Iris*_	86.36	86.36	0.838	5.568
		*B*_*Iris*_	86.36		0.837	

## 5 Discussion

In our proposed work, the vertical collaborative clustering using bit plane slicing approach is studied and applied over all eight-bit planes per feature of Geyser, Skin and Iris datasets. Since there are 2, 3 and 4 features in Geyser, Skin and Iris data at site A and B respectively, therefore, the numbers of bit plane combinations are 64 (8^2^), 512 (8^3^) and 4096 (8^4^) respectively. The findings reveal BP (5,6) and (7,6,7) as the most significant bits combinations for Geyser and Skin data, whereas BP (6,2,1,2) as both most and least combinations for Iris data, capturing similarity. These bit plane combinations have such important bits which capture contrast between given class labels as the least or most significant bits or both to correctly classify class labels at particular bit plane with the least misclassification. They have high purity with good compactness (DB) value in comparison to existing approaches. It is not necessary that the collaborative purity will always be the mean of the local purities for all methods. For instance, our proposed approach (VCC-BPS) returns exact mean of local purities but existing approaches return collaborative purity not exactly equal to the mean of the local purities. The reason behind such symmetric and asymmetric collaborative purity is that the existing approaches have topographic map of fix size having nodes to represent similar observations. These nodes may be surplus and do not participate to represent data or have the least data observations at one site in match to another site. This deteriorates the final map results once K-mean algorithm is applied [4]. As a result, the collaborative purity corresponding to the existing approaches is asymmetric. Therefore, the collaborative purity measured at site A is different from that at B. Our proposed approach is very effective to deal with such problem by considering only those code maps which participate to capture similarity locally and collaboratively, and discard other code maps which do not participate. This forms symmetry, giving collaborative purity as the mean of the local purities.

This study shows that 120 training observations of the Geyser data at site A and B each, are compressed into 4 code maps (2 bits per code map) locally and collaboratively at bit plane (5,6). In case of Iris data, the contrast among three given class labels is captured and 66 training observations at site A and B are compressed into 14 code maps (4 bits per code map) and 2 code maps (0001 and 1101) do not participate in data compression locally and collaboratively at bit plane (6,2,1,2). Likewise, using same approach for Skin dataset, contrast between two given class labels is captured and more than 98000 training observations at site A and B each, are compressed into 8 code maps locally and collaboratively at bit plane (7,6,7).

The proposed collaborative purity reflects the similarity among the participating sites as high if the difference between the local and collaborative purity is low and vice versa. Moreover, if the local purity is less than the collaborative purity, means local learning enhanced by collaboration and accordingly collaborative DB increases based on Eq (7). Such increase in collaborative DB confirms similarity between respective clusters of different data sites, whereas the low local DB shows the quality clustering within the local data. The Table 7 shows the out-performance of the proposed approach in comparison to the existing approaches with quality clustering in terms of increased purity and collaborative DB with high test data accuracy. Notably, the bit plane at which an optimal solution is obtained, varies from dataset to dataset. Moreover, if the dataset with large number of features is used then the accuracy will not be compromised but computational cost will increase. Additionally, the collaborative purity results are closer to the global purity, verifies accuracy of our proposed approach. It also reveals that the proposed approach is successful to capture distributed hidden behavior which is similar to that of pooled dataset.

## 6 Conclusion and future work

This paper presents vertical collaborative clustering using bit plane slicing to manage collaboration among different sites. In this novel approach, an adequate common bit plane is determined among participating data sites, at which model fits the data with maximum similarity to unlock hidden patterns. Investigation shows that there is at least one-bit plane which captures relative important information commonly shared among different data sites. Notably, the bit planes, which contribute the most to represent relative important information, vary from dataset to dataset. The comparison of the proposed with the existing approaches reveals that VCC-BPS outperforms by having superior accuracy in term of high purity with improved DB and compress a large number of observations into smaller code space. The proposed collaborative results are close to that of pooled data output which verifies its accuracy. However, the proposed approach has a vast search space finding bit planes with an adequate solution for a dataset with large feature space. This requires further investigation to add an extra computational layer such as using a data compression technique before voting algorithm to unravel the most informative bit plane and reduce the computational cost of measuring similarity both locally and collaboratively.

## Supporting information

S1 File (ZIP)
