# Supplementary figures and images for: VCC-BPS: Vertical Collaborative Clustering using Bit Plane Slicing
Source: PLoS One. 2021 Jan 11;16(1):e0244691. doi: 10.1371/journal.pone.0244691 (PMC7799819; doi:10.1371/journal.pone.0244691)

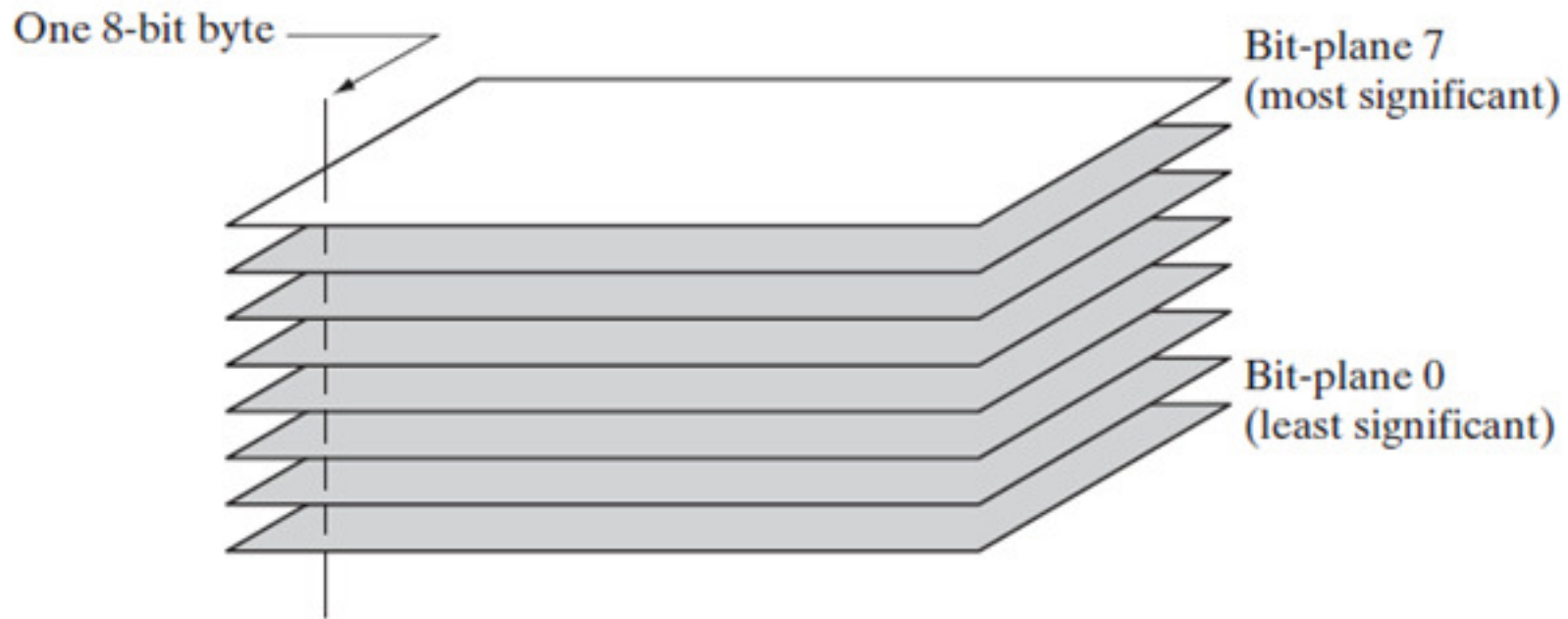

Supplement: S1 File — (ZIP) [file pone.0244691.s001.zip › Manuscript/Bit_Plane_Slicing1-eps-converted-to.pdf]

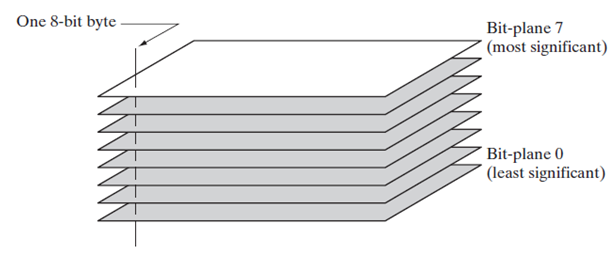

Supplement: S1 File — (ZIP) [file pone.0244691.s001.zip › Manuscript/Bit_Plane_Slicing1.png]

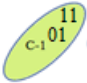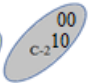

Supplement: S1 File — (ZIP) [file pone.0244691.s001.zip › Manuscript/BP_36-eps-converted-to.pdf]

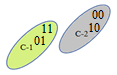

Supplement: S1 File — (ZIP) [file pone.0244691.s001.zip › Manuscript/BP_36.png]

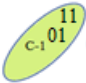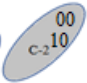

Supplement: S1 File — (ZIP) [file pone.0244691.s001.zip › Manuscript/BP_46-eps-converted-to.pdf]

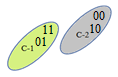

Supplement: S1 File — (ZIP) [file pone.0244691.s001.zip › Manuscript/BP_46.png]

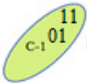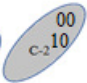

Supplement: S1 File — (ZIP) [file pone.0244691.s001.zip › Manuscript/BP_56-eps-converted-to.pdf]

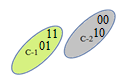

Supplement: S1 File — (ZIP) [file pone.0244691.s001.zip › Manuscript/BP_56.png]

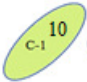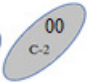

Supplement: S1 File — (ZIP) [file pone.0244691.s001.zip › Manuscript/BP_57-eps-converted-to.pdf]

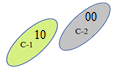

Supplement: S1 File — (ZIP) [file pone.0244691.s001.zip › Manuscript/BP_57.png]

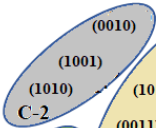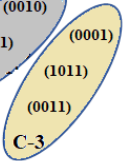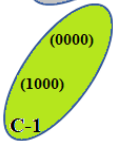

Supplement: S1 File — (ZIP) [file pone.0244691.s001.zip › Manuscript/BP_5712-eps-converted-to.pdf]

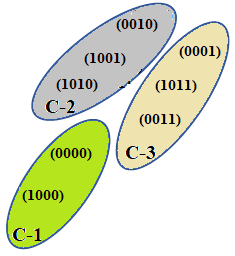

Supplement: S1 File — (ZIP) [file pone.0244691.s001.zip › Manuscript/BP_5712.png]

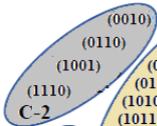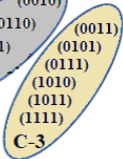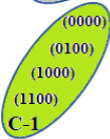

Supplement: S1 File — (ZIP) [file pone.0244691.s001.zip › Manuscript/BP_6212-eps-converted-to.pdf]

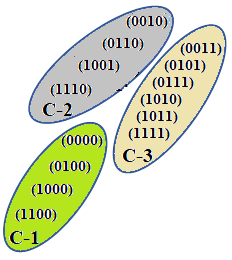

Supplement: S1 File — (ZIP) [file pone.0244691.s001.zip › Manuscript/BP_6212.png]

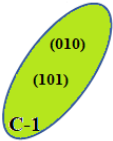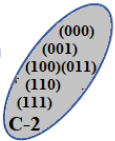

Supplement: S1 File — (ZIP) [file pone.0244691.s001.zip › Manuscript/BP_668-eps-converted-to.pdf]

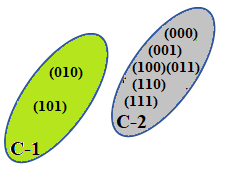

Supplement: S1 File — (ZIP) [file pone.0244691.s001.zip › Manuscript/BP_668.png]

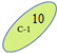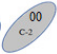

Supplement: S1 File — (ZIP) [file pone.0244691.s001.zip › Manuscript/BP_67-eps-converted-to.pdf]

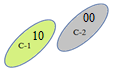

Supplement: S1 File — (ZIP) [file pone.0244691.s001.zip › Manuscript/BP_67.png]

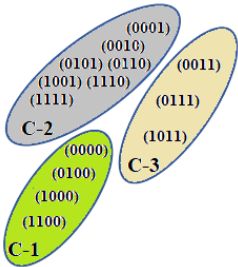

Supplement: S1 File — (ZIP) [file pone.0244691.s001.zip › Manuscript/BP_7312-eps-converted-to.pdf]

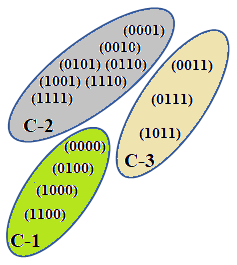

Supplement: S1 File — (ZIP) [file pone.0244691.s001.zip › Manuscript/BP_7312.png]

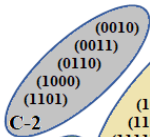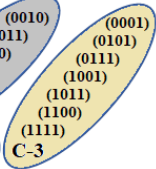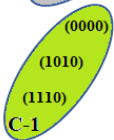

Supplement: S1 File — (ZIP) [file pone.0244691.s001.zip › Manuscript/BP_7452-eps-converted-to.pdf]

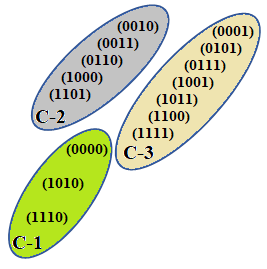

Supplement: S1 File — (ZIP) [file pone.0244691.s001.zip › Manuscript/BP_7452.png]

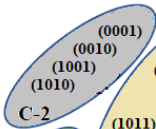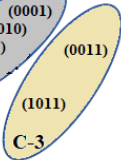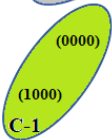

Supplement: S1 File — (ZIP) [file pone.0244691.s001.zip › Manuscript/BP_7612-eps-converted-to.pdf]

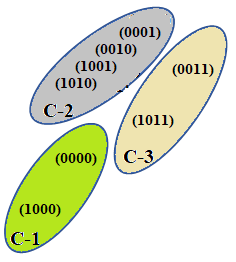

Supplement: S1 File — (ZIP) [file pone.0244691.s001.zip › Manuscript/BP_7612.png]

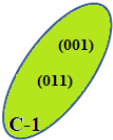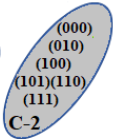

Supplement: S1 File — (ZIP) [file pone.0244691.s001.zip › Manuscript/BP_878-eps-converted-to.pdf]

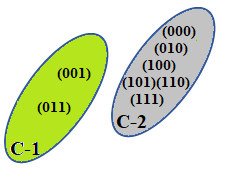

Supplement: S1 File — (ZIP) [file pone.0244691.s001.zip › Manuscript/BP_878.png]

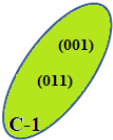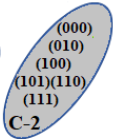

Supplement: S1 File — (ZIP) [file pone.0244691.s001.zip › Manuscript/BP_888-eps-converted-to.pdf]

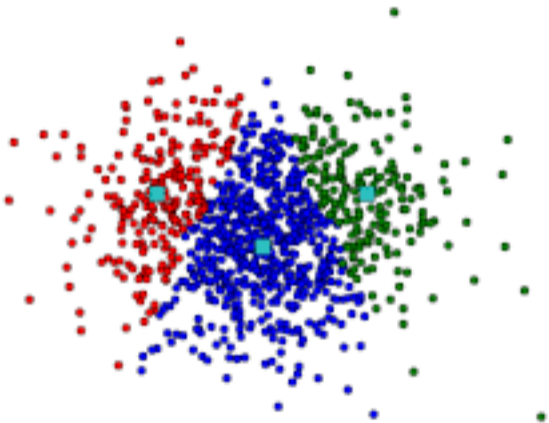

Supplement: S1 File — (ZIP) [file pone.0244691.s001.zip › Manuscript/Fig_1-eps-converted-to.pdf]

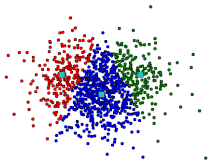

Supplement: S1 File — (ZIP) [file pone.0244691.s001.zip › Manuscript/Fig_1.png]

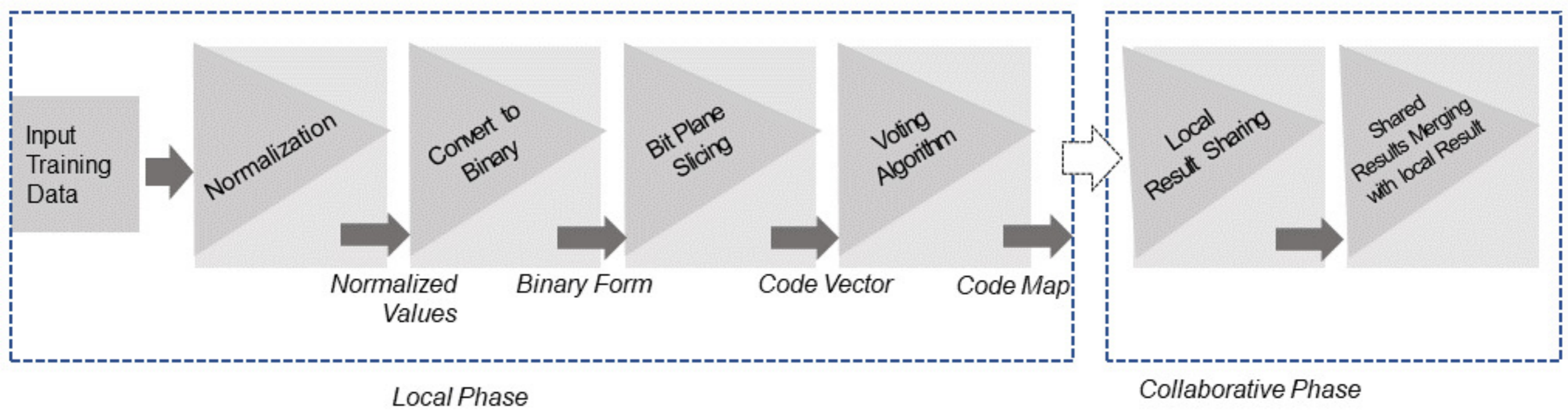

Supplement: S1 File — (ZIP) [file pone.0244691.s001.zip › Manuscript/media/Block_diag_fig_3-eps-converted-to.pdf]

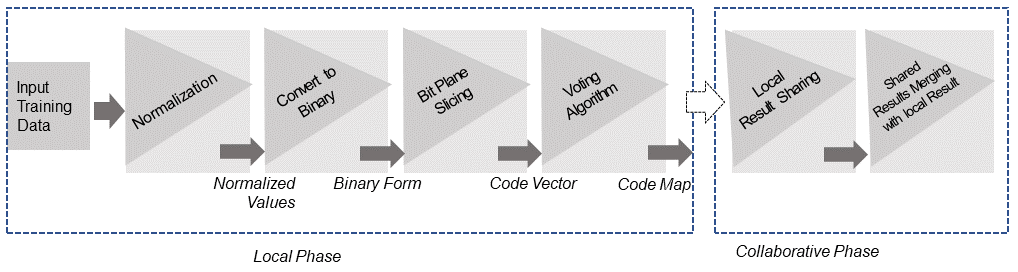

Supplement: S1 File — (ZIP) [file pone.0244691.s001.zip › Manuscript/media/Block_diag_fig_3.png]

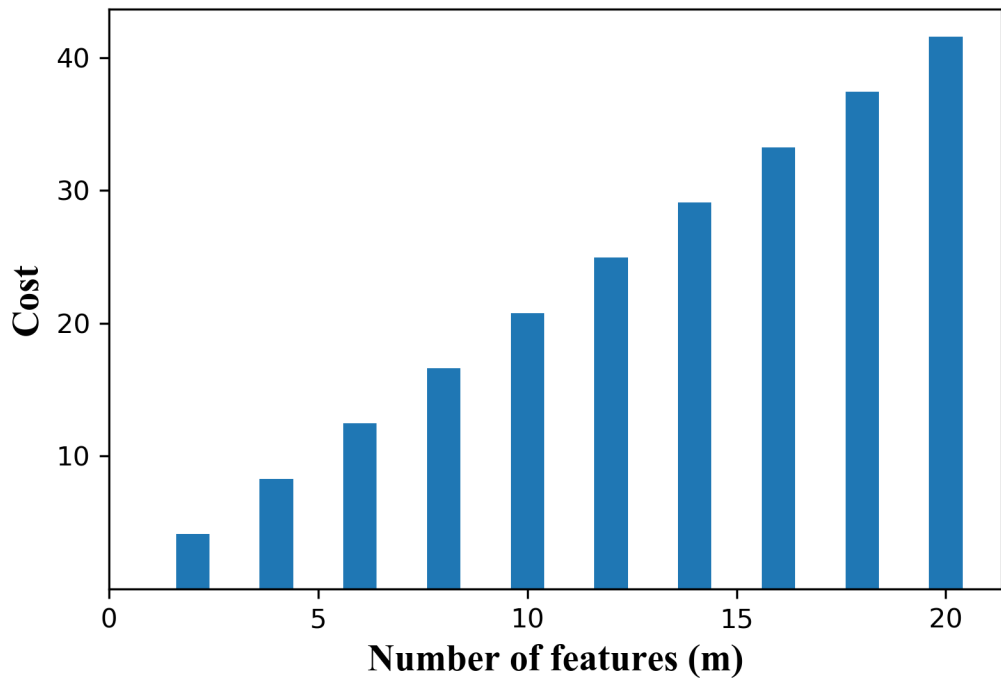

Supplement: S1 File — (ZIP) [file pone.0244691.s001.zip › Manuscript/media/myGraph-eps-converted-to.pdf]

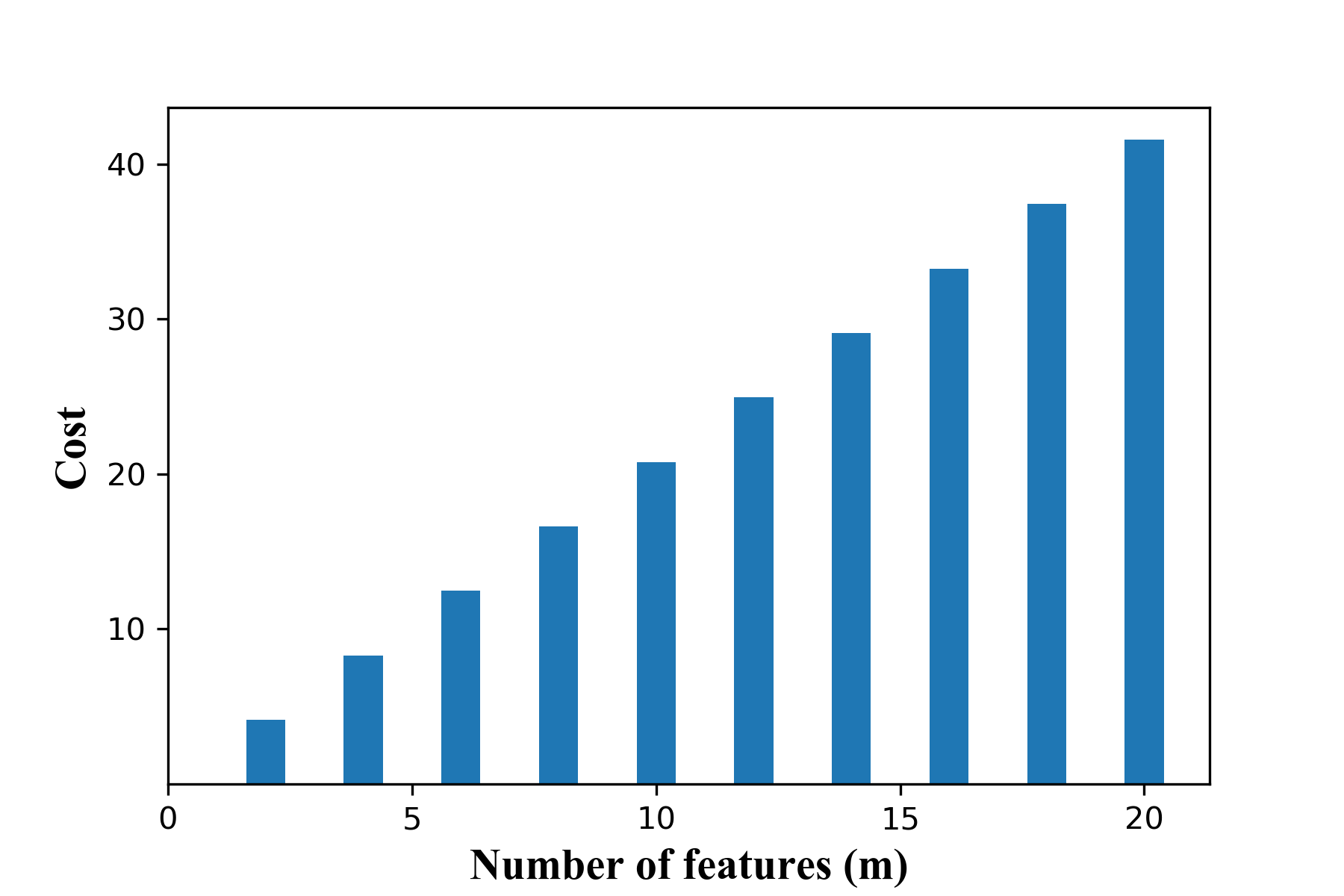

Supplement: S1 File — (ZIP) [file pone.0244691.s001.zip › Manuscript/media/myGraph.png]

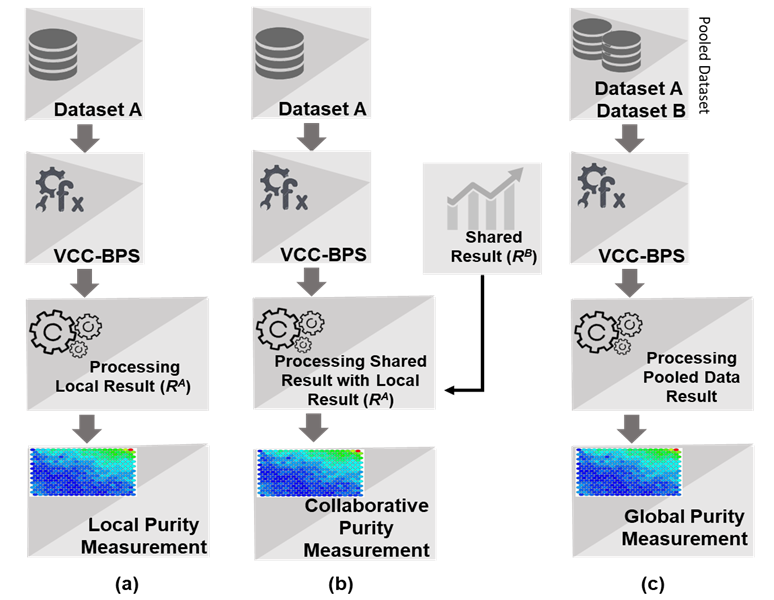

Supplement: S1 File — (ZIP) [file pone.0244691.s001.zip › Manuscript/media/Purity_figure_5.png]

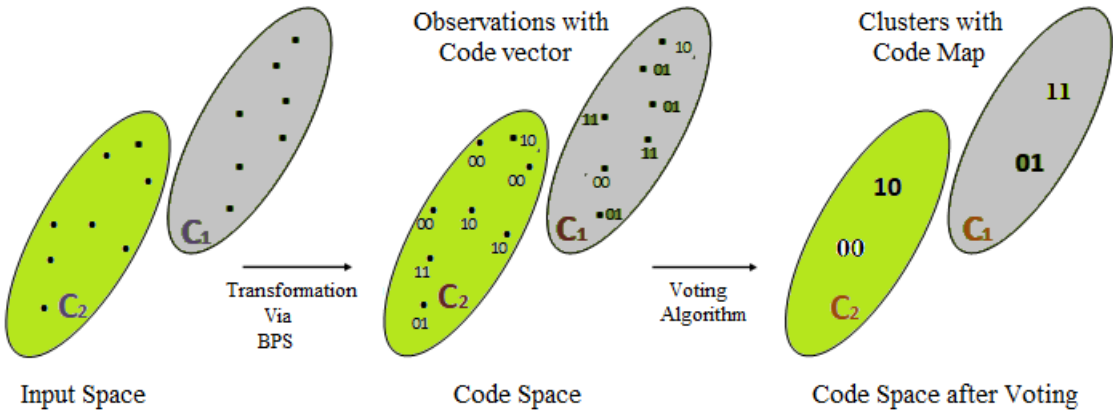

Supplement: S1 File — (ZIP) [file pone.0244691.s001.zip › Manuscript/media/Transform_fig_2-eps-converted-to.pdf]

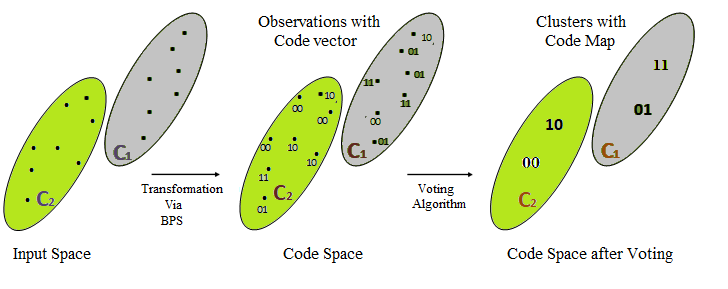

Supplement: S1 File — (ZIP) [file pone.0244691.s001.zip › Manuscript/media/Transform_fig_2.png]

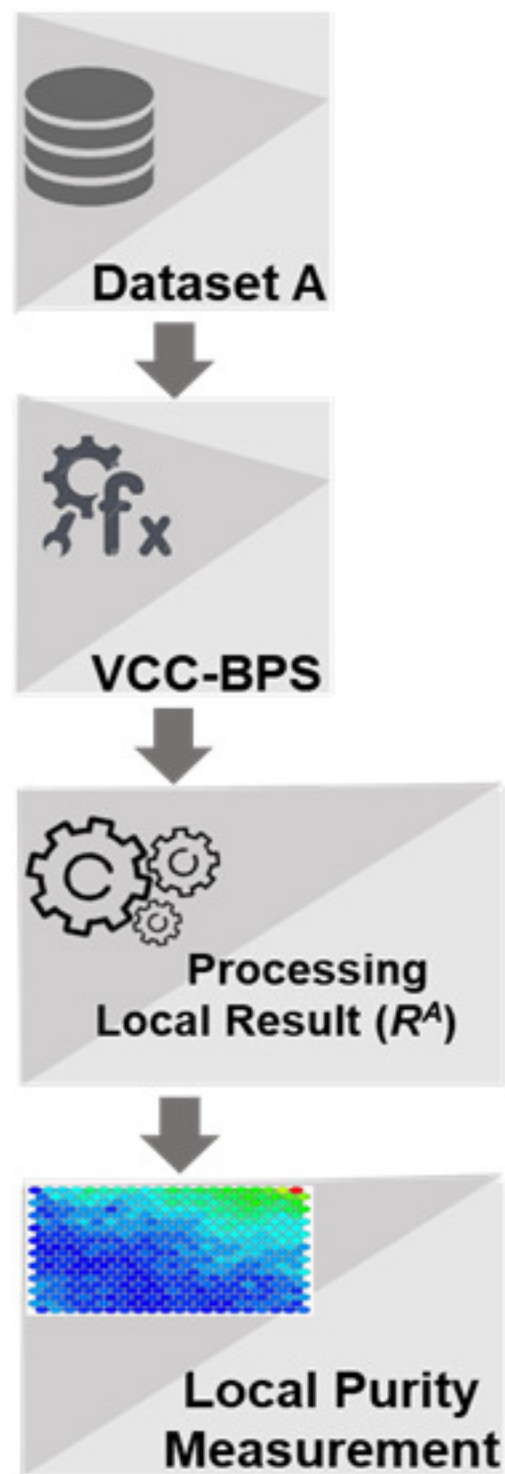

(a)

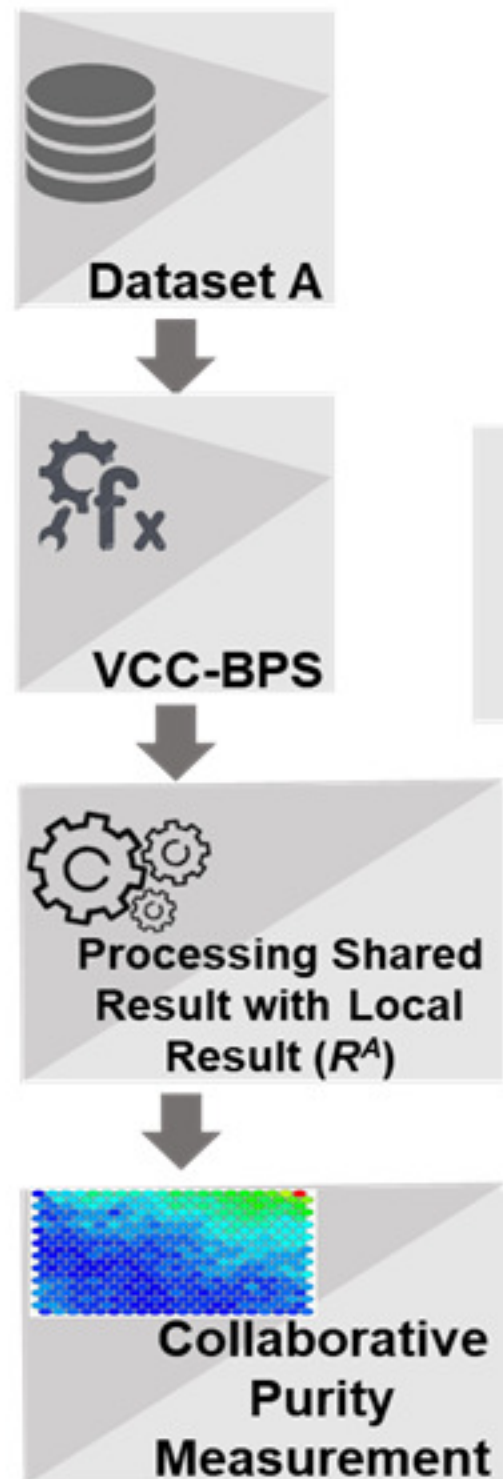

(b)

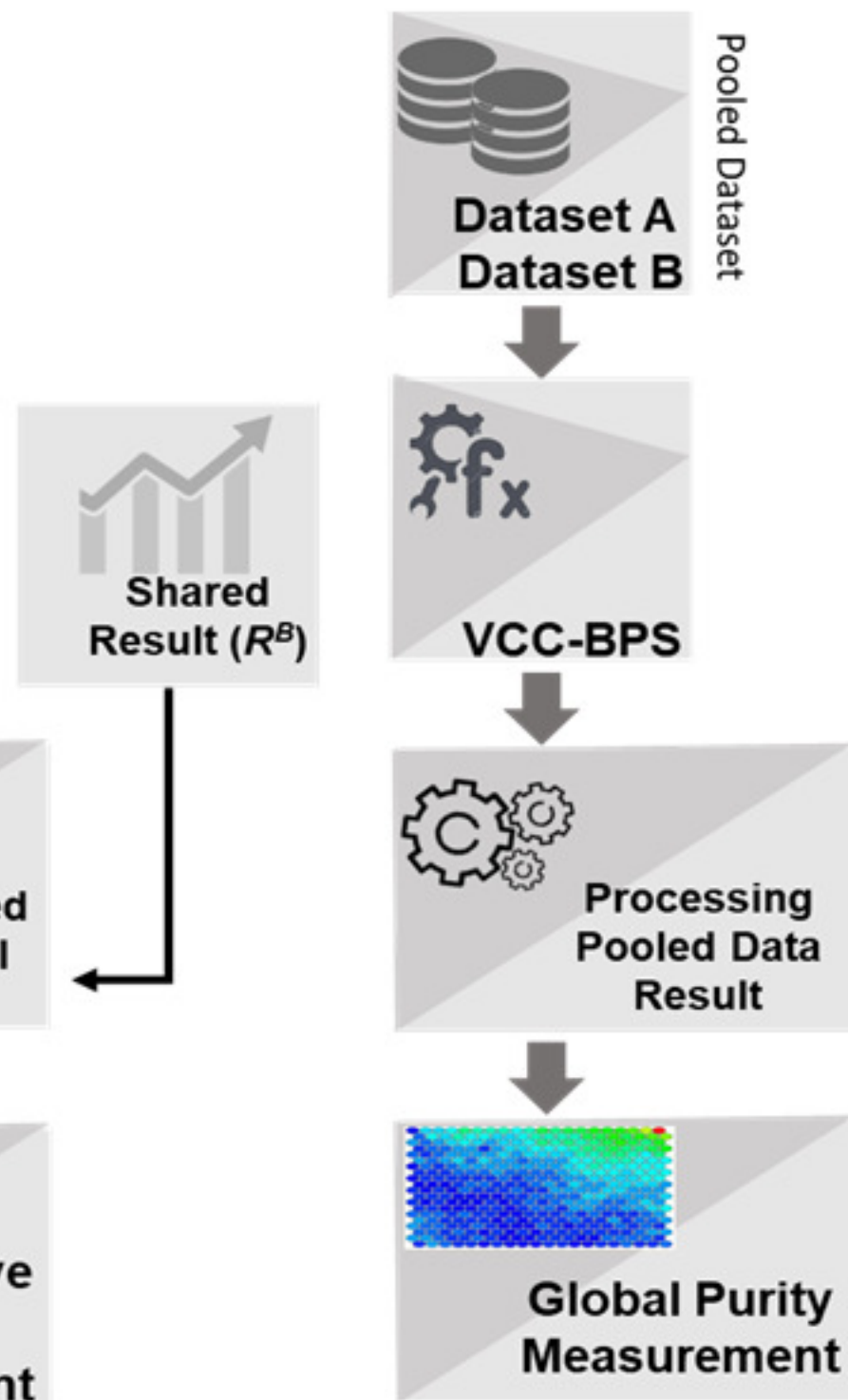

(c)

Supplement: S1 File — (ZIP) [file pone.0244691.s001.zip › Manuscript/Purity_figure_5-eps-converted-to.pdf]

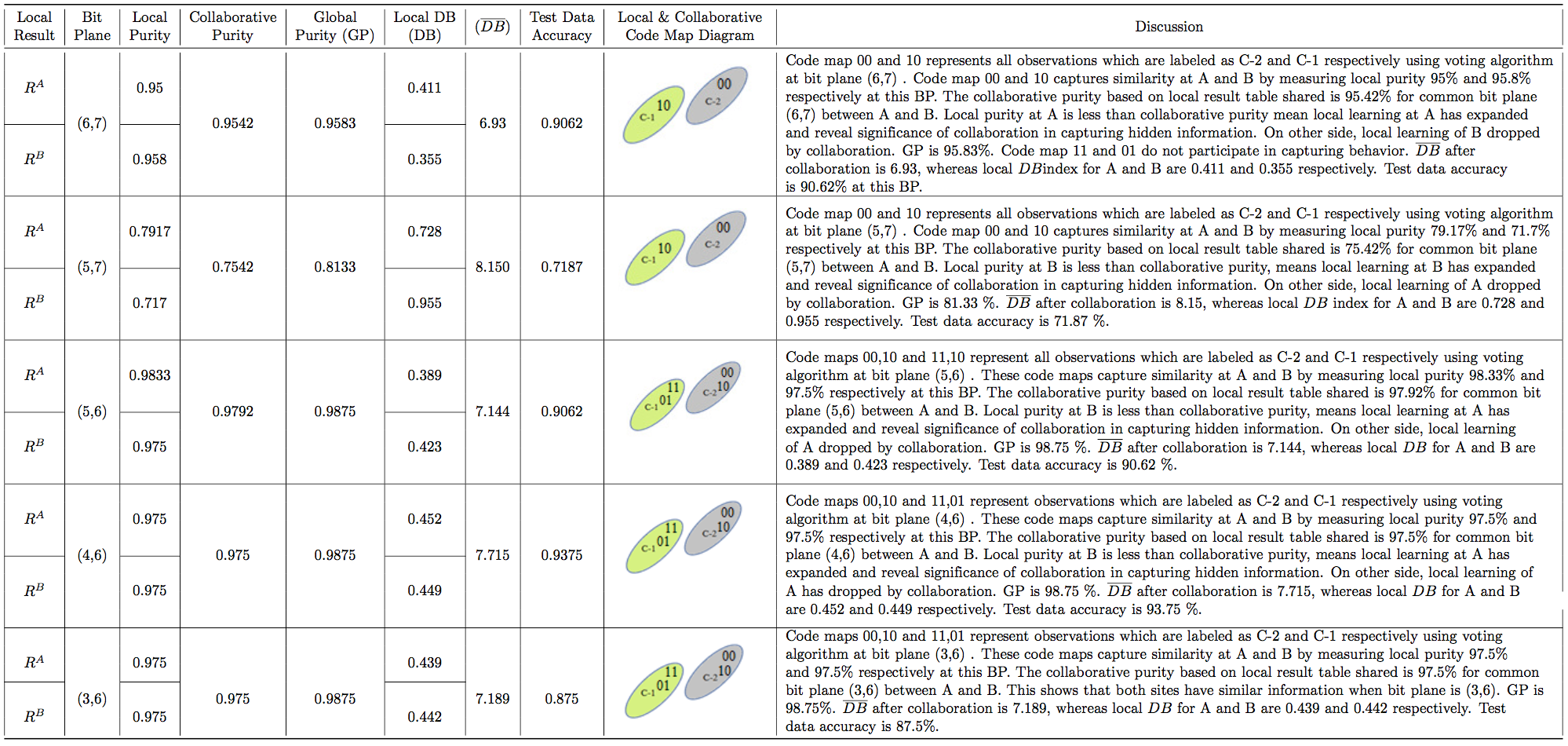

Supplement: S1 File — (ZIP) [file pone.0244691.s001.zip › Manuscript/Tables789/Table7.png]

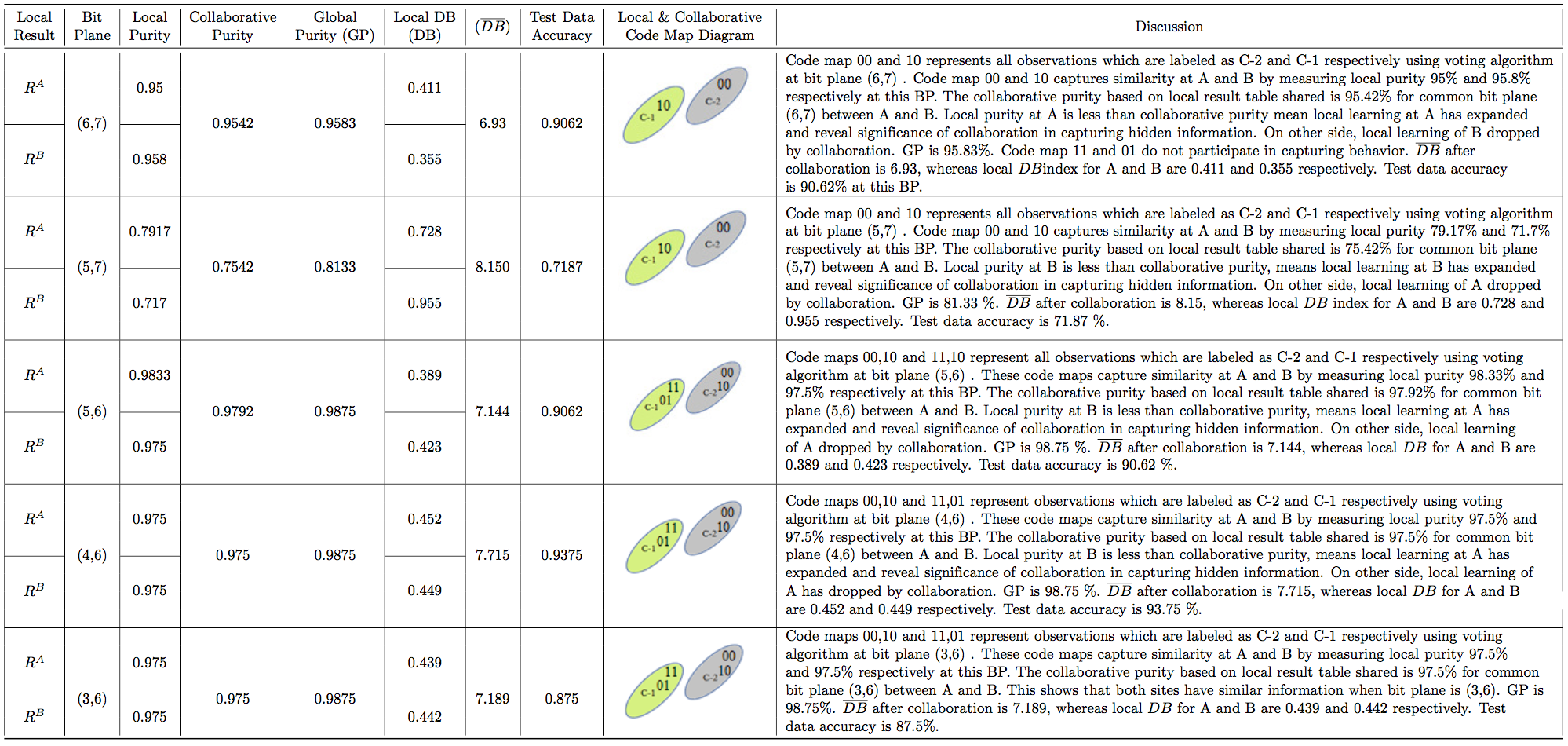

Supplement: S1 File — (ZIP) [file pone.0244691.s001.zip › Manuscript/Tables789/Table7.tif]

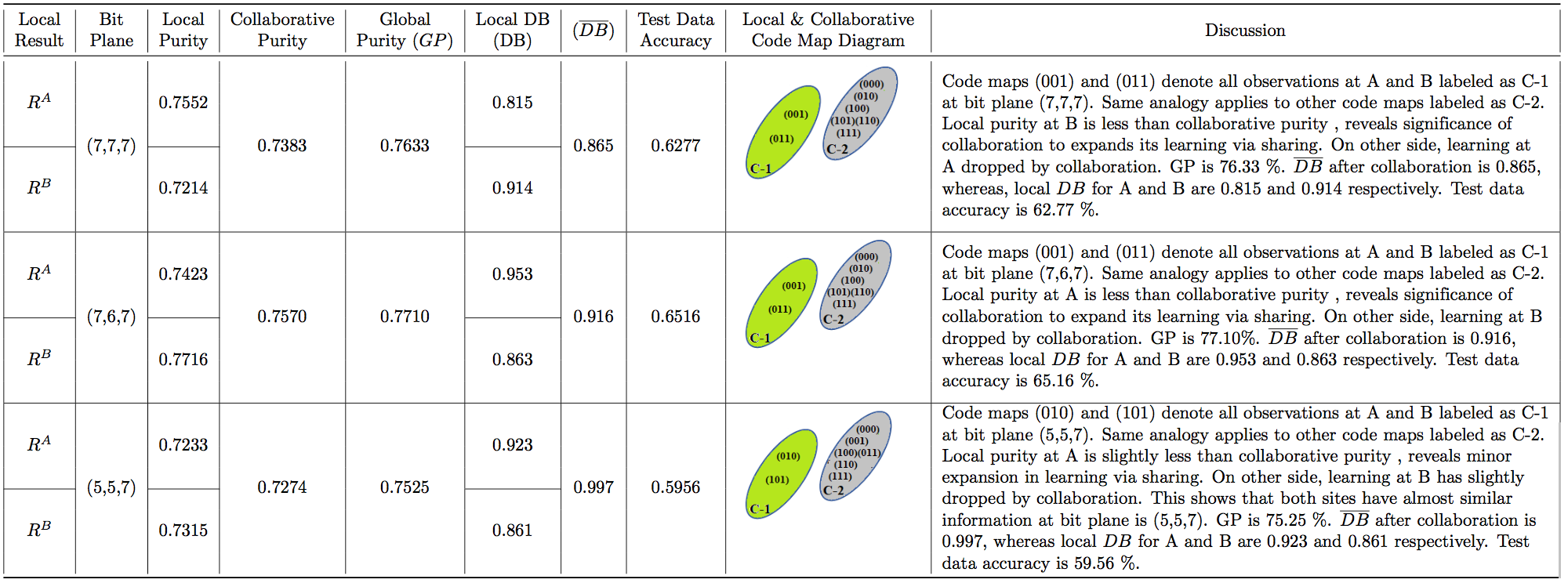

Supplement: S1 File — (ZIP) [file pone.0244691.s001.zip › Manuscript/Tables789/Table8.png]

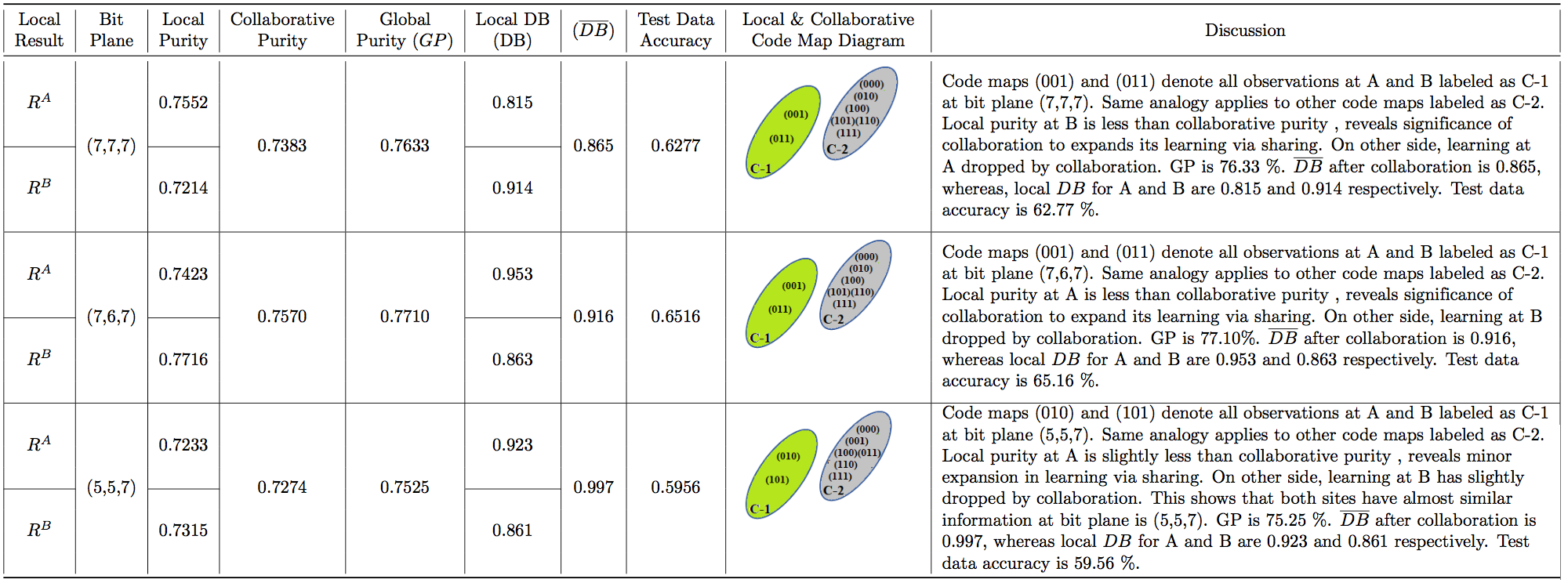

Supplement: S1 File — (ZIP) [file pone.0244691.s001.zip › Manuscript/Tables789/Table8.tif]

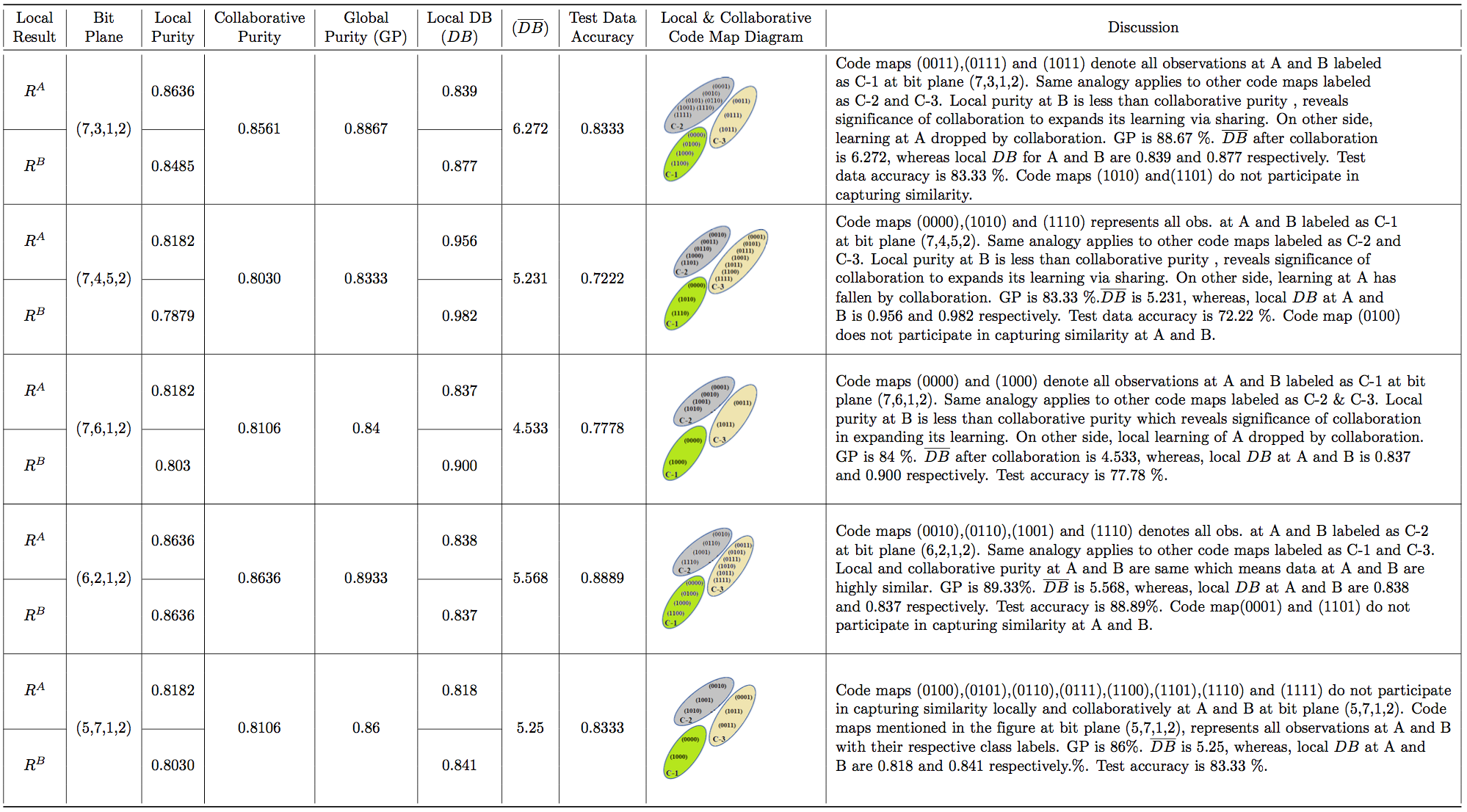

Supplement: S1 File — (ZIP) [file pone.0244691.s001.zip › Manuscript/Tables789/Table9.png]

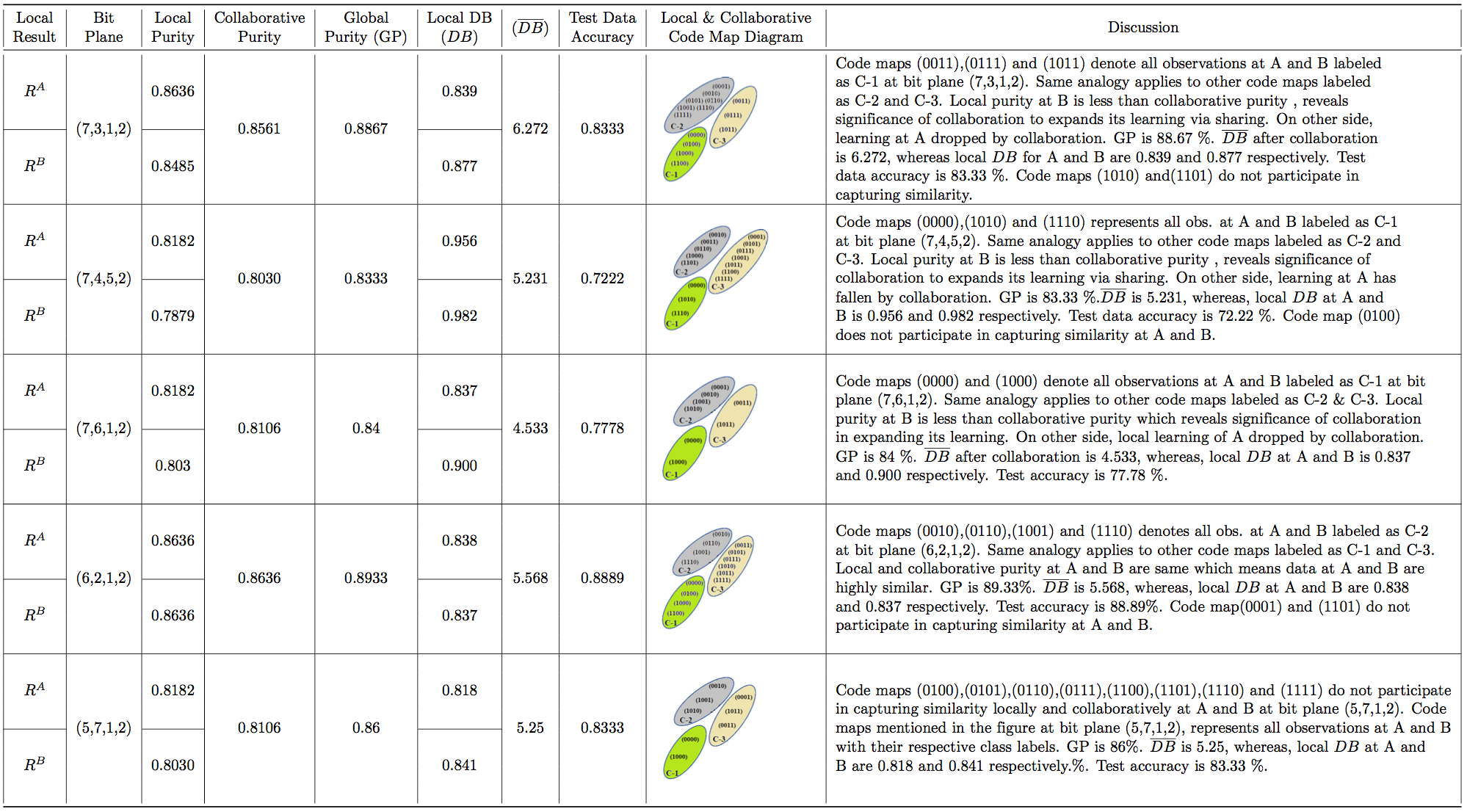

Supplement: S1 File — (ZIP) [file pone.0244691.s001.zip › Manuscript/Tables789/Table9.tif]
